# Bacteriophages benefit from generalized transduction

**DOI:** 10.1371/journal.ppat.1007888

**Published:** 2019-07-05

**Authors:** Alfred Fillol-Salom, Ahlam Alsaadi, Jorge A. Moura de Sousa, Li Zhong, Kevin R. Foster, Eduardo P. C. Rocha, José R. Penadés, Hanne Ingmer, Jakob Haaber

**Affiliations:** 1 Institute of Infection, Immunity and Inflammation, University of Glasgow, Glasgow, United Kingdom; 2 Department of Veterinary and Animal Sciences, University of Copenhagen, Frederiksberg, Denmark; 3 Microbial Evolutionary Genomics, Institut Pasteur, CNRS, UMR3525, Paris, France; 4 CAS Center for Excellence in Molecular Plant Sciences, Shanghai Institute of Plant Physiology and Ecology, Chinese Academy of Sciences, Shanghai, China; 5 Department of Zoology, University of Oxford, Oxford, United Kingdom; 6 Department of Biochemistry, University of Oxford, Oxford, United Kingdom; National Institutes of Health, UNITED STATES

## Abstract

Temperate phages are bacterial viruses that as part of their life cycle reside in the bacterial genome as prophages. They are found in many species including most clinical strains of the human pathogens, *Staphylococcus aureus* and *Salmonella enterica* serovar Typhimurium. Previously, temperate phages were considered as only bacterial predators, but mounting evidence point to both antagonistic and mutualistic interactions with for example some temperate phages contributing to virulence by encoding virulence factors. Here we show that generalized transduction, one type of bacterial DNA transfer by phages, can create conditions where not only the recipient host but also the transducing phage benefit. With antibiotic resistance as a model trait we used individual-based models and experimental approaches to show that antibiotic susceptible cells become resistant to both antibiotics and phage by i) integrating the generalized transducing temperate phages and ii) acquiring transducing phage particles carrying antibiotic resistance genes obtained from resistant cells in the environment. This is not observed for non-generalized transducing temperate phages, which are unable to package bacterial DNA, nor for generalized transducing virulent phages that do not form lysogens. Once established, the lysogenic host and the prophage benefit from the existence of transducing particles that can shuffle bacterial genes between lysogens and for example disseminate resistance to antibiotics, a trait not encoded by the phage. This facilitates bacterial survival and leads to phage population growth. We propose that generalized transduction can function as a mutualistic trait where temperate phages cooperate with their hosts to survive in rapidly-changing environments. This implies that generalized transduction is not just an error in DNA packaging but is selected for by phages to ensure their survival.

## Author summary

Viruses (phages) that only attack bacteria are highly common. Some of these phages naturally reside within the bacterial chromosome for extended periods of time. Upon release and propagation on phage susceptible cells, new phage particles are made but occasionally bacterial rather than phage DNA is packaged into phage heads. Transfer of random pieces of bacterial DNA between cells by such transducing particles has been termed generalized transduction and has long been used as a tool for genetic engineering. We show here that bacteria and generalized transducing phages cooperate to survive adverse conditions such as the presence of antibiotics by phage integration and transduction of antibiotic resistance genes from neighboring cells. The resulting cells are resistant to phage attack due to immunity provided by the integrated prophage and they are able to exchange bacterial DNA with other cells containing a similar prophage via the transducing particles. Previously, transduction has been considered an accident in phage biology. Here we propose that rather than being an accident, generalized transduction is an evolved trait selected by some temperate phages to persist in rapidly-changing environments.

## Introduction

Temperate bacteriophages (phages) have a dual life cycle. They reside in the bacterial chromosome as prophages until induction initiates lytic replication, where phage structural proteins are produced, phage DNA is packaged into virions, and the cell ultimately lyses releasing the phage progeny. Prophages are common in bacteria and almost half of the bacterial genomes carry prophages with pathogens more likely being lysogens than non-pathogens [1]. For the Gram-positive, human pathogen, *Staphylococcus aureus*, essentially all clinical strains carry between 1 and 4 prophages [2] and for the Gram-negative pathogen, *Salmonella enterica* serovar Typhimurium, prophages are present in the majority of strains [3,4]. While prophage induction obviously can reduce host viability through lytic replication [5,6] and negatively impact host fitness either by providing a metabolic cost [3] or by disrupting host genes upon integration [4,7], there are also examples of mutualistic interactions between temperate phages and their hosts [8]. Foremost, prophages provide immunity to attack from related phages by expressing the phage repressor protein that controls the transition between temperate and lytic replication [5,9]. They can also express a variety of adaptive accessory genes [8,10], and for bacterial pathogens, prophage encoded virulence factors contribute to colonization and pathogenesis [4,8,11]. Despite these mutalistic interactions, phages are largely considered parasites in a continous arms race with the host [12] where the constant attack from phages have led to the evolution of an impressive arsenal of anti-phage systems [13].

One example where bacteria have been thought to benefit from phages is transduction. In this process bacterial DNA is packaged into phage particles and is transferred between bacterial cells. In organisms such as *S*. *aureus*, transduction is considered to be the major contributor to the spread of antibiotic resistance genes and the success of the pathogen [2,14]. Transduction can take place in various forms. In specialized transduction, DNA flanking the prophage attachment site (*att*B) is transferred as a consequence of an aberrant prophage excision. In the recently discovered lateral transduction, late excision and *in situ* replication of an integrated prophage leads to highly efficient packaging of bacterial DNA several hundred kilobases downstream of the integration site [15]. Finally, in generalized transduction phages randomly package bacterial DNA instead of their own and thus, can essentially transfer any piece of the bacterial genome [16]. Generalized transduction is mediated by temperate phages that employ the *pac* site–headful mechanism for DNA packaging [16] as opposed to the *cos*-phages which package DNA of exactly one genome delimited by *cos* sites [17]. After being discovered, generalized transduction soon became a powerful genetic tool, which at the time revolutionized microbial genetics [18] [19]. Early on it was also observed that the transductants, namely the cells receiving bacterial DNA, often carry a copy of the phage in their genome thus becoming lysogens [20–22]. This made it unclear whether the transduced bacterial DNA was transferred independently or in functional phage particles [22,23]. Subsequent studies demonstrated that viral particles mediating generalized transduction contain bacterial DNA and are non-functional from a phage perspective [24]. Based on these observations, transduction has for many years been considered to be a consequence of errors in the phage DNA packaging machinery allowing bacterial rather than phage DNA to be packaged [4,16,25,26].

Recently we found that lysogens can acquire genes from non-lysogens by infecting them with phages that transfer back these genes to the original population by transduction [9]. We have continued to study the role of transduction in phage-host interactions and propose here that infection of a bacterial cell by a temperate phage and a transducing particle can increase fitness of both by allowing the host cell to acquire adaptive genes, whose benefits are shared with the prophage. This creates a direct association between transduction and phage fitness that could explain the existence of high rates of transduction by certain phages. Using *S*. *aureus* and *S*. *enterica* Typhimurium as experimental models and *in silico* modelling, we show that under a certain range of conditions, generalized transduction provides the phage with bet-hedging opportunities that increase its own and the hosting cells chances of surviving in changing environments. These findings indicate that transduction is an intrinsic part of phage biology and that transducing phages benefit from the process by providing adaptive power to the hosting bacterium.

## Results

### Transduction is linked to lysogenization

**Fig 1:**
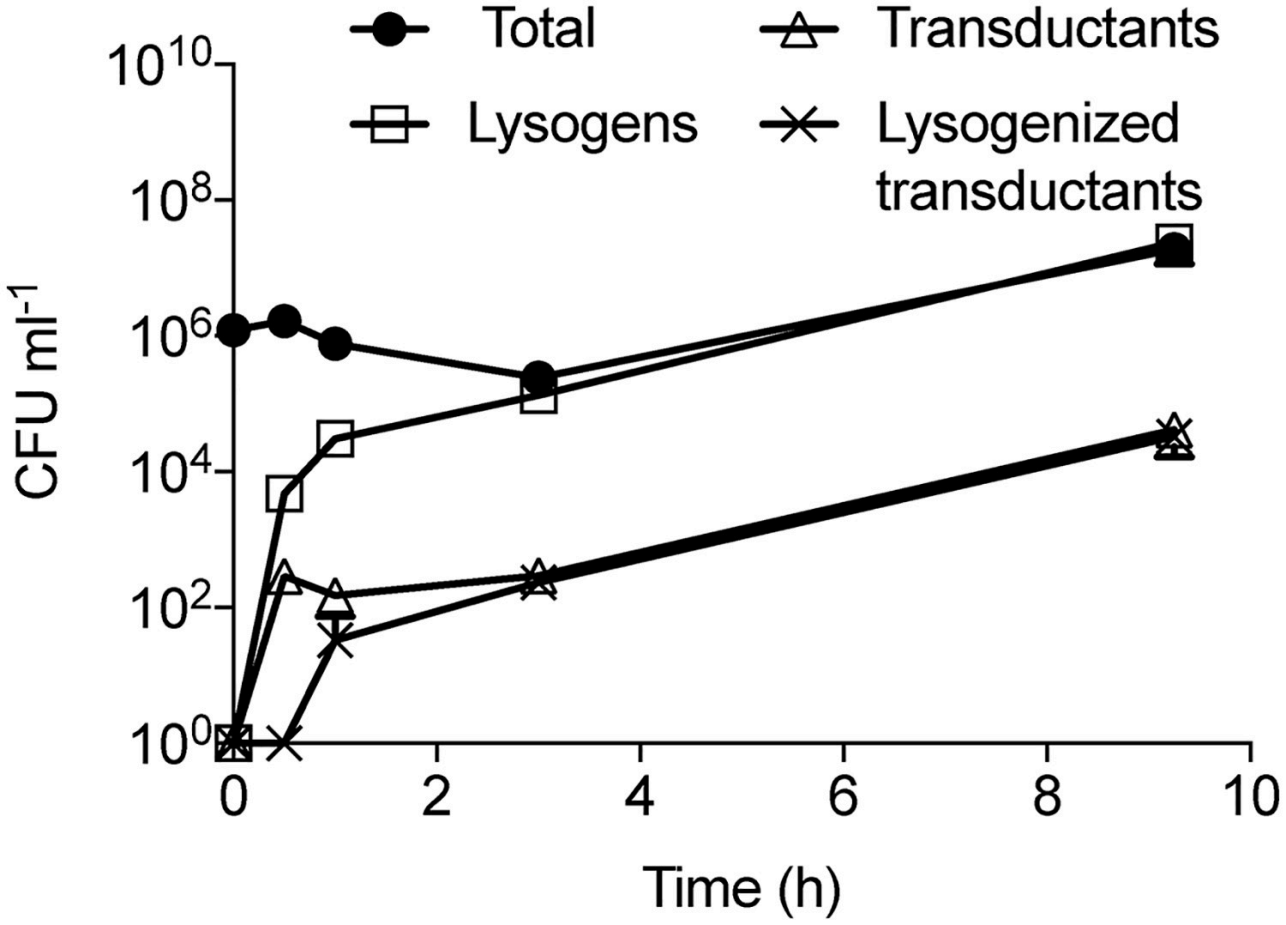
Time course experiment of *S*. *aureus* infected with ϕ11-ERM transducing lysate. At time 0, *S*. *aureus* cells (RN4220) were infected at MOI = 1 with ϕ11-ERM propagated on JH930 harboring the pRMC2 plasmid encoding chloramphenicol resistance. At regular intervals (1, 3 and 9h), the total number of cells was determined as was the number of transductants (being resistant to chloramphenicol) and the number of transductants being lysogens and thus resistant to erythromycin.

In a previous study we observed that lysogens of *S*. *aureus* carrying the generalized transducing phage ϕ11 were able to acquire DNA from non-lysogenic cells following spontaneous phage release in a process we termed autotransduction [9]. To address more generally what happens when phages, propagated on antibiotic resistant cells, meet bacteria that are neither resistant to antibiotics nor carry prophages, we examined a variety of phages infecting either *S*. *aureus*, *S*. *enterica* serovar Typhimurium or *E*. *coli*. In these experiments we monitored generalized transduction as the phage population used for infection was prepared by propagation on antibiotic resistant cells rather than by induction of a lysogen. At a multiplicity of infection (MOI) of 1 and an initial cell density of 0.01 (OD_600_), the number of colony forming units, CFU, transductants and lysogens were determined following overnight incubation. For the generalized transducing and temperate *S*. *aureus* phages ϕ11, ϕ52A, ϕ53 and 80α and *Salmonella enterica* serovar Typhimurium phage P22, transductants formed with a frequency of 10^3^ to 10^4^ CFU/ml and essentially all (95–100%) were lysogens (Table 1). In contrast, no transductants were observed with the non-generalized transducing *E*. *coli cos*-type temperate phage λ, the lytic *S*. *aureus* phage Sa012 or the *S*. *aureus* 80α-vir, a virulent derivative of the transducing 80α phage. Despite the lack of transductants for both *S*. *aureus* phage Sa012 and *S*. *aureus* 80α-vir, the phage lysates contained transducing particles packed with bacterial DNA as determined by PCR (S1 Fig). When monitoring over time the infection with a derivative of ϕ11 encoding resistance to erythromycin (ϕ11-ERM) propagated on chloramphenicol resistant cells, the bacterial culture after 3 hours was already dominated by lysogens (10^5^ cells) and of these, 10^2^ were transductants (Fig 1). These results show that the survival of transductants depends on lysogeny providing resistance to infection by similar phages, at least under the examined conditions where the phage infection process is not limited.

**Table 1:** Transduction and lysogenization in cells when infected with temperate phages (upper part) or lytic phages (lower part).

Phage	Recipient strain	Transductants (CFU/ml)	Total (CFU/ml)	Percent lysogenic transductants*
*S*. *aureus* ϕ52a	RN4200	1.6E+04 ± 3.8E+03	5.7E+09 ± 3.9E+09	100
*S*. *aureus* ϕ53	RN4220	3.2E+04 ± 1.2E+04	2.3E+10 ±1.0E+10	95
*S*. *aureus* ϕ11	RN4220	2.9E+04 ± 1.1E+04	3.1E+10 ± 1.1E+10	99
*S*. *aureus* ϕ80a	RN4220	3.4E+04 ± 2.1E+04	3.2E+10 ± 1.3E+10	100
*Salmonella* P22	LT2	2.7E+03 ± 2.5E+03	3.4E+08 ± 2.7E+08	100
*E*. *coli* lambda	C600	< 10	4.8E+07 ± 3.6E+07	ND
*S*. *aureus* ϕ Sa012	RN4220	< 10	1.0E+09 ± 1.9E+08	ND
*S*. *aureus* ϕ 80a-vir	RN4220	< 10	8.4E+08 ± 7.5E+08	ND
no phage control	RN4220	< 10	3.5E+10 ± 2.5E+09	ND

* Percent lysogens calculated from total CFU.

### Modelling transduction and lysogeny in changing environments

**Fig 2:**
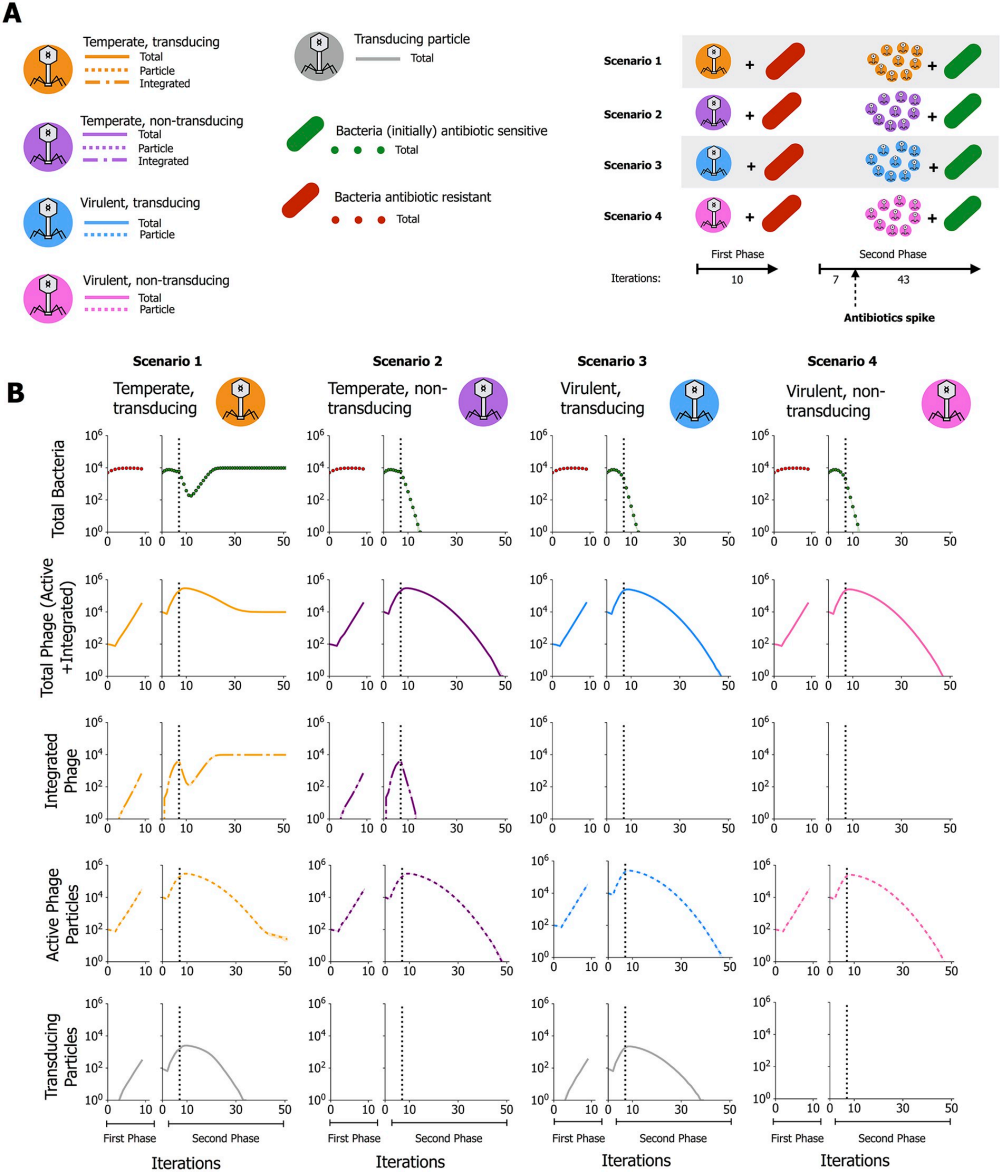
Simulations of three different experimental setups involving infection by one single type of phage show an advantage of transducing temperate phages in changing environments. **A)** Phages infect bacteria carrying an antibiotic resistance gene (bacteria in red), and a sample of these phage particles infects bacteria initially susceptible to antibiotics (in green). Antibiotics are applied after the initial 7 iterations. 4 different scenarios are explored, using different types of phages: temperate and transducing phages in orange; temperate, and non-transducing phages in purple; virulent and transducing phages in blue; and virulent and non-transducing phages in pink. **B)** The number of bacteria (antibiotic resistant bacteria in red, and–initially–antibiotic sensitive bacteria in green) and the different types of phage particles are followed over time for the 4 different scenarios (each scenario corresponds to a column). The two different phases of the experiment (infection of antibiotic resistant bacteria and subsequently infection of antibiotic sensitive bacteria) are indicated in the x-axis. Lines correspond to the median of 100 different simulations with similar parameters, and the shaded areas correspond to a confidence interval of 95%. The model used in the simulations, as well as the values assumed for the parameters, are detailed in S1 Text.

We used an individual-based model to understand how generalized transduction and lysogeny affects population outcome when there is selection for a transduced marker. The framework, eVIVALDI [27], simulates populations composed of discrete individual organisms reproducing and dying in a lattice. Each individual has a number of attributes and behaviours, including explicit genomes. These types of models are suited for making qualitative analyses of a complex system where many different variables can potentially affect its outcome. Population-level dynamics emerge from the interactions among these individuals and with their environment (for a recent review see [28]). Here, we defined a community with bacteria and phages, and explored various scenarios with different types of phages (Fig 2A). Bacteria reproduce and compete for space in the lattice whereas phages reproduce by infecting bacteria. The complete model, including parameters and their explanation, is specified in the ODD (Overview, Design concepts, and Details) protocol of the model provided as S1 Text. In an initial phase (first phase, first 10 iterations), the phages infected bacteria carrying the antibiotic resistance marker. A sample of the virions including generalized transducing particles was then introduced to a population of antibiotic sensitive bacteria (second phase), and a dose of antibiotics was added at the indicated time point. Initially, when the phage was temperate and a transducer (Fig 2B, first column, scenario 1), the bacterial population decreased because of phage predation. When antibiotics were added to the environment, only populations of bacteria that acquired the antibiotic resistance gene were predicted to have survived. First the phage population increased as a result of replication of virulent and temperate forms of the phage, and subsequently decreased matching the dwindling bacterial populations. Individuals in our model have explicit genomes, and transduction results in genomic changes. As expected, the analysis of the genomes of the surviving bacteria predicted them to carry a prophage (i.e. they become lysogens) and an antibiotic resistance gene (S2 Fig). At the end of the simulations all bacteria were lysogens (they are protected from phages) and carried the antibiotic resistance gene (were infected by a transducing particle). By this time, almost all phages were in the state of prophages, and the number of free phages is dictated only by the rate of spontaneous prophage induction.

We also simulated the use of temperate but non-generalized transducing phages (Fig 2C, second column, scenario 2). In this case, the dynamics were the same up to the antibiotic spike, after which the bacterial population was predicted to become extinct by the combined effect of phage predation and antibiotics. The lack of host cells led to the extinction of the phage population. When the phages were virulent, and capable of generalized transduction (Fig 2B, scenario 3), the bacterial population decreased following propagation of the phage. The spike of antibiotics led to the extinction of the population in all simulations. This fits the expectation that if bacteria cannot become lysogens, and thus are not protected from lytic phage infections, transduction cannot help them survive. Similar results were obtained using virulent non-generalized transducing phages (Fig 2B, scenario 4). Overall, in 100 replicate simulations for each scenario, both bacteria and phage survived in nearly all (98%) of the replicates in scenario 1, and always went extinct in scenarios 2, 3 and 4. In conclusion, phages only survive if they are lysogens and transducers. This shows that transduction favours phage survival.

**Fig 3:**
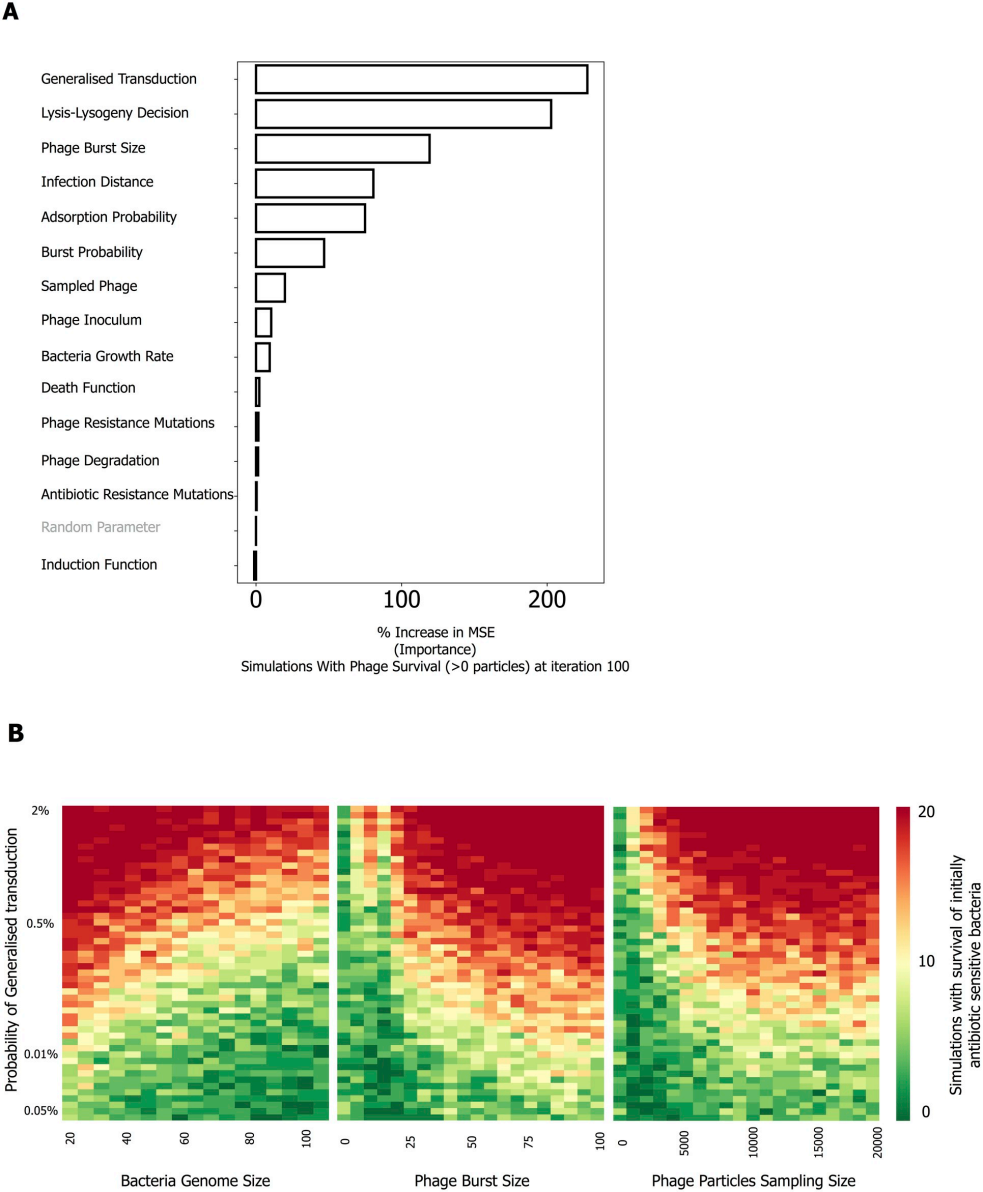
Identification of relevant parameters for phage survival. **A)** Random Forest Analysis (RFA) is based on 5000 randomized combinations of parameter values and 20 repeated simulations for each combination. Parameters with a higher % in increased minimum square error have greater impact on the measured outcome: the fraction of simulations (for the 20 replicate simulations with a similar parameter combination) where phage are found alive. The parameters varied in the RFA are shown in S1 Text, section 3. **B)** Detailed effects of 3 variables in the survivability of initially antibiotic sensitive bacteria. The outcomes of simulations are shown in function of the probability of generalized transduction (y-axis) and genome size of the antibiotic resistant bacteria on which the phage was propagated (first panel), phage burst size (second panel) and number of phage particles sampled from the first part of the experiment (third panel) used for the second part of the experiment (see Fig 2). Each bin corresponds to the median of 20 replicate simulations for each parameter combination.

The model is parameter-rich and these parameters are not always easy to define. This raises questions on their biological relevance and on how their variation can affect the final outcome. The parameters for these simulations (see Section 2 of S1 Text) were empirically set to understand whether the joint role of transduction and lysogeny could lead to long-term (i.e., after 100 iterations) phage survival in these conditions. Since other parameters could also play a role in these dynamics, we performed an unbiased combinatorial Random Forest Analysis (RFA) of 5000 different combinations of parameters that contribute to phage survival. Each combination of parameters was used in 20 different simulations, for a total of 100,000 simulations (see Methods). The ranges of parameters explored are indicated in S1 Text (Section 3). In general, we aimed at having broad ranges of parameters that allow a diversity of outcomes. The analysis indicates that the probability of generalized transduction is the most influential parameter for phage survival (either as lysogens or active particles), followed by the probability of lysogenization (Fig 3A). The results are robust to variations in the other parameters (S3 Fig). We then explored in further detail the likelihood of bacterial survival, which in this system is equivalent to phage survival as bacterial survivors are lysogens and essentially all phages are prophages. Here the survival was assessed as a function of the probability of generalized transduction and two of the variables explored in the RFA (phage burst size and the number of sampled phage between the first and second phase), as well as an additional one, namely the size of the bacterial genome on which the phage had been propergated. As seen in Fig 3B, first panel, the frequency of antibiotic resistant transductants is predicted to decrease with increasing genome size because the probability that the virion carries the bacterial antibiotic resistance gene decreases with increasing bacterial genome size. In contrast, survival increases with phage burst size because larger bursts are predicted to result in more transducing particles, thus increasing the probability of transfer of the resistance gene (Fig 3B, second panel). Finally, survival is calculated to increase when the number of phage particles sampled between the experimental steps is initially increased and then makes a plateau, because a very low sample size may lack a transducing particle with the resistance gene (Fig 3B, third panel). All these analyses show that transduction of the antibiotic trait occurs within a wide, but defined, region of parameters. Overall, the model fits the notion that transduction and lysogeny jointly facilitate the acquisition of the adaptive trait. In particular, our results suggest that infection by temperate phages capable of forming lysogens, and their transducing particles, enable survival of the phages (and their hosts) in a dynamic environment exposed to antibiotics, when the transfer of resistance genes is possible.

### Transduction and lysogeny confer survival in changing environments

**Fig 4:**
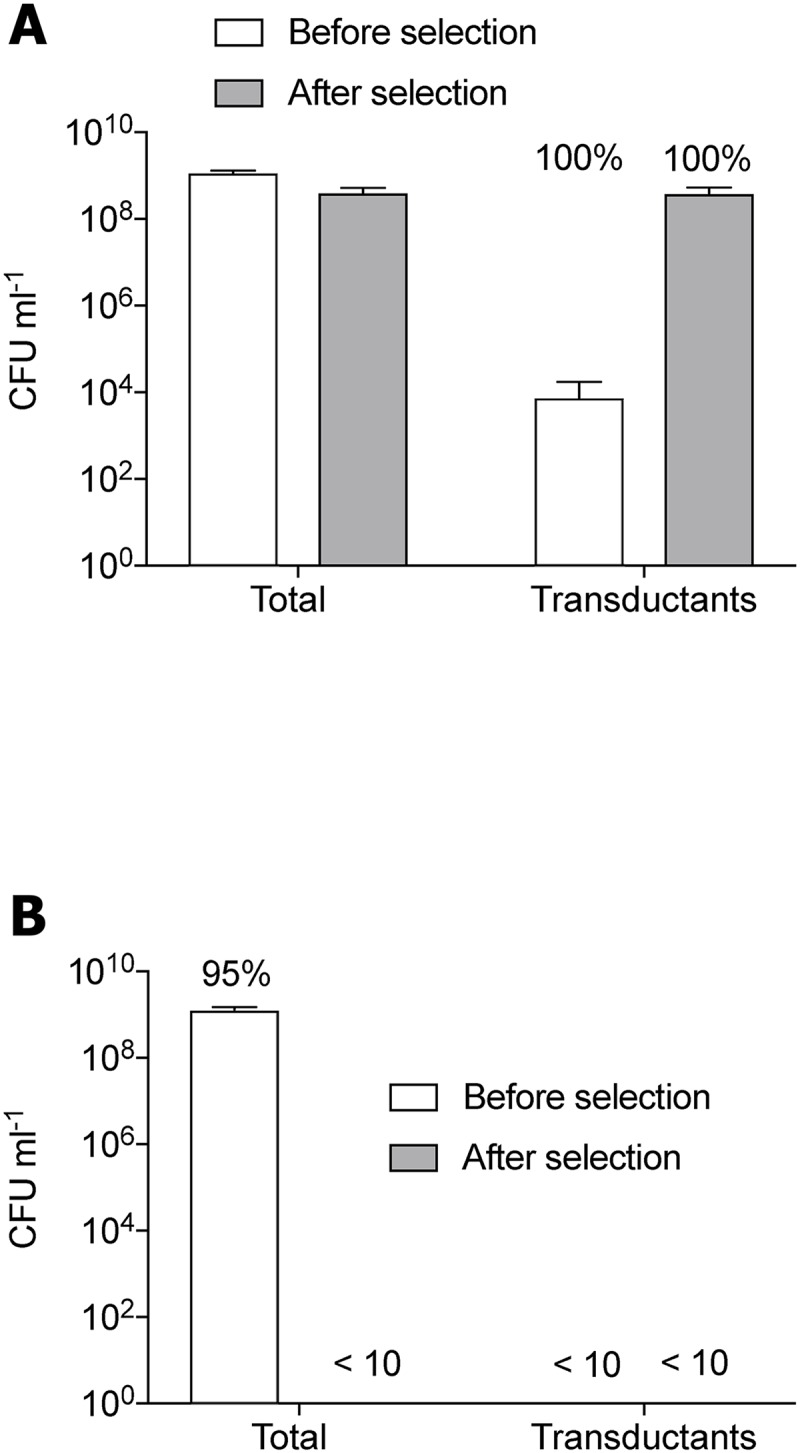
Generalized transduction (GT) allows phage to persist in the presence of antibiotics. RN4220 was infected with GT *pac* phage, ϕ11 (**A**) or non-GT, *cos*-phage ϕ12 (**B**) propagated on strain JH930 harboring pRMC2 encoding resistance to chloramphenicol. After 8 generations of growth the total number of bacteria and the number of chloramphenicol-resistant transductants were determined. Then, each culture was diluted 1000 fold in media containing a lethal concentration (30μg/ml) chloramphenicol and grown for 8 generations. The dilution step was repeated and the cultures were allowed to grow for additional 8 generations after which CFU of all cultures was determined on plates with or without chloramphenicol. Hundred transductants (or just survivors in case of ϕ12) were tested for lysogeny. Percentage over graphs indicate percentage of lysogens of total number of colonies tested.

To experimentally examine the role of transduction in phage survival under changing environments we compared the fate of two *S*. *aureus* temperate phages namely the generalized transducing ϕ11 and the non-generalized transducing (*cos*-type) ϕ12 (not to be confused with the lytic ϕSa012) before and after antibiotic exposure. Both phages were propagated on a chloramphenicol resistant strain and the resulting phage lysates were used to infect phage-susceptible cells. After 8 generations of growth in non-selective media (Fig 4A, before selection) chloramphenicol was added and growth was followed (Fig 4A, after selection). In the culture infected with the generalized transducing ϕ11 phage, the CFU was approximately 1x10^9^ ml^-1^ before addition of chloramphenicol and of these cells, 1x10^4^ ml^-1^ were transductants (being resistant to chloramphenicol) and lysogens (100%) (Fig 4A). Following growth in the presence of chloramphenicol, the transductants propagated and reached 5x10^8^ CFU ml^-1^ with 100% of the cells being lysogens. In contrast, and as predicted, for the culture infected with the non-generalized transducing ϕ12 phage, no chloramphenicol-resistant transductants were observed neither before nor after selection (Fig 4B). Before antibiotic exposure, ninety-five percent of the cell population infected with ϕ12 were lysogens demonstrating that ϕ12 was able to lysogenize its host. However, after selection, the antibiotic had eliminated bacteria and thus the prophage. This experiment confirms that the generalized transducing ability of ϕ11 enables the phage and the hosting lysogen to survive in the presence of antibiotic exposure when transducing particles are available with the observed trait.

**Fig 5:**
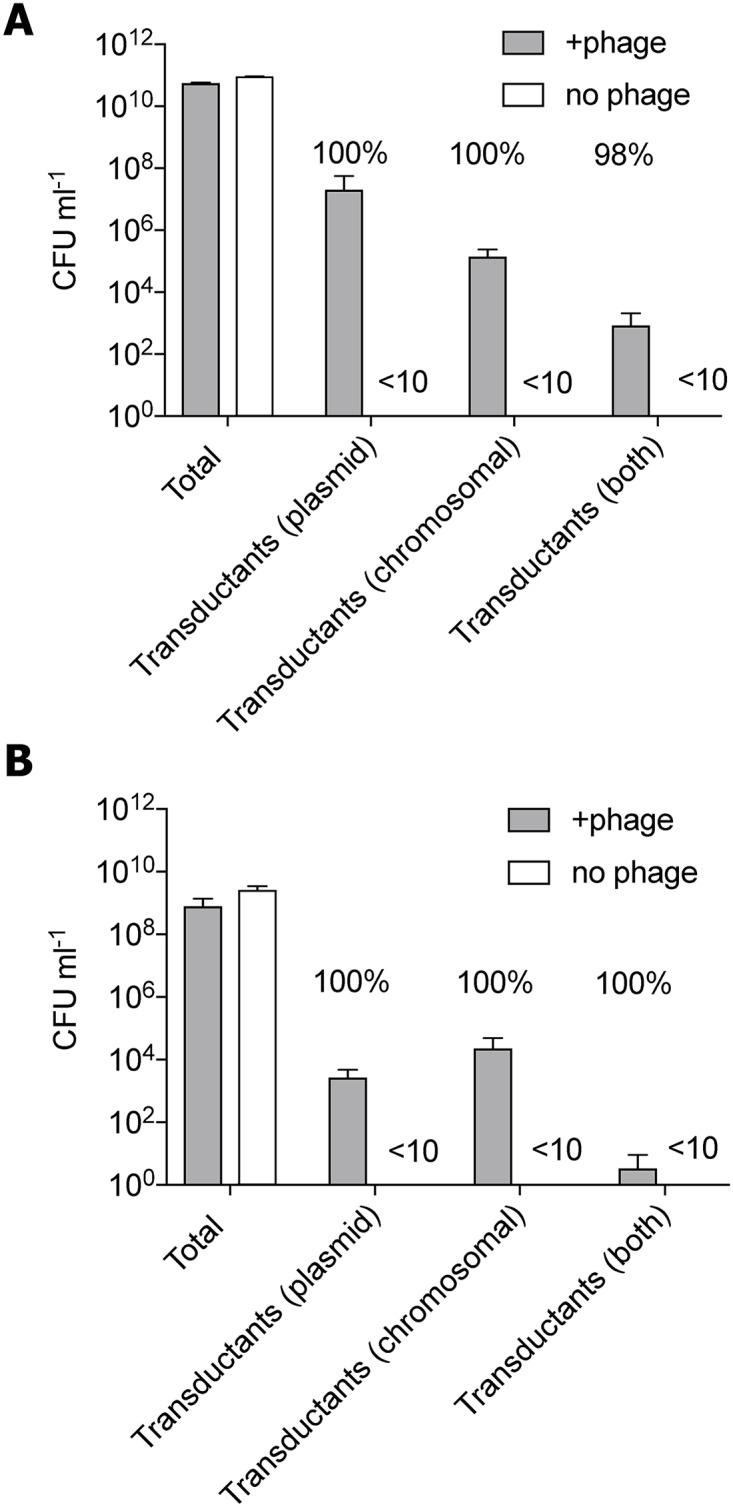
Efficient transduction transfers unlinked plasmid and chromosomal markers. **(A)** *S*. *aureus* RN4220 after overnight infection with a ϕ11-ERM lysate propagated on a donor strain (JH1064) containing pRMC2 plasmid (CmR30) and a chromosomal marker (TetR5) inserted in the *agr* operon. **(B)** *S*. *enterica* Typhimurium LT2 after overnight infection with a phage P22 lysate propagated on a donor strain (JP14365) containing plasmid pET28a (KmR30) and a chromosomal marker (TetR20) inserted into the *prp*R gene. In both cases bar diagrams represent CFU ml^-1^ after incubation on selective plates. Grey bars represent phage infected cultures and white bars represent non-infected control cultures. From 66 to 150 transductants in each category were tested for lysogeny. Numbers above graphs indicate percentage of lysogens of total number of colonies tested.

We further explored the adaptive potential of transduction by investigating the transfer of two unlinked genetic markers. We engineered strains of *S*. *aureus* and *S*. *enterica* Typhimurium to contain chromosomal and plasmid-encoded antibiotic resistance markers. Phage lysates (ϕ11-ERM and P22, respectively) obtained by propagation on these strains were used to infect phage-susceptible cells and after overnight incubation the number of transductants was determined. The result shows that both plasmid and chromosomal markers were transferred by transduction in *S*. *aureus* (Fig 5A) and *S*. *enterica* Typhimurium (Fig 5B). Notably, some recipient cells (approximately 10^3^) were resistant to both the plasmid and the chromosomally-encoded markers, showing that within the time frame of the experiment, two unrelated markers were transduced into a single cell. Again, the majority (98–100%) of the transductants were lysogenized by the transducing phage (Fig 5A and 5B) demonstrating that bacteria and phages alike benefit from the transduction process.

To address the capacity of transducing particles to carry bacterial DNA we used quantitative real time (qRT-PCR) to detect the chloramphenicol resistance gene in a ϕ11 phage lysate propagated on *S*. *aureus* carrying pRMC2 expressing chloramphenicol resistance and found that the transducing particles constituted 1 in every 700 infective phage particles in the lysate as determined as described by Varga et al. [29] (S1 Table) corroborating other studies reporting approximately 1 out of 1000 *S*. *aureus* phages to be a generalized transducing particle [30]. Our *S*. *aureus* phage lysates contain 1x10^9^ PFU ml^-1^ and thus approximately 1.5x10^6^ transducing particles ml^-1^. As each of these particles can harbour up to 43 kbp of bacterial DNA [31] each millilitre of a phage lysate can carry up to 61 billion bp bacterial DNA, the equivalent of more than 20,000 full *S*. *aureus* genomes. Importantly this number is an average estimate based on the information on the number of generalized transducing particles. If the phage lysates was generated by induction of a lysogen the frequency of genes downstream of the integration site in the viral particles is likely to be much higher due to lateral transduction [15]. These numbers underline the potential for transduction to move large amounts of bacterial DNA between cells. In summary, our results show that transduction linked to lysogenization allows the phage to efficiently generate genetic variation in a population of host bacteria that helps the phage and its bacterial host to acquire novel genes required for survival in changing environments.

### Phages mediate bacterial evolution

In the previous experiments, high titer phage lysates obtained on strains carrying transducible antibiotic resistance markers were used to infect phage-susceptible recipient cells. Under natural conditions the generalized transducing phages are released from the lysogens following induction, for example by DNA damaging agents, or in smaller numbers by spontaneous release [9] [32] [33]. This suggests that during co-cultivation, genetic material can be exchanged between different lysogens (carrying the same or related prophages) through the induction of their prophages, without significant lysis being observed.

**Fig 6:**
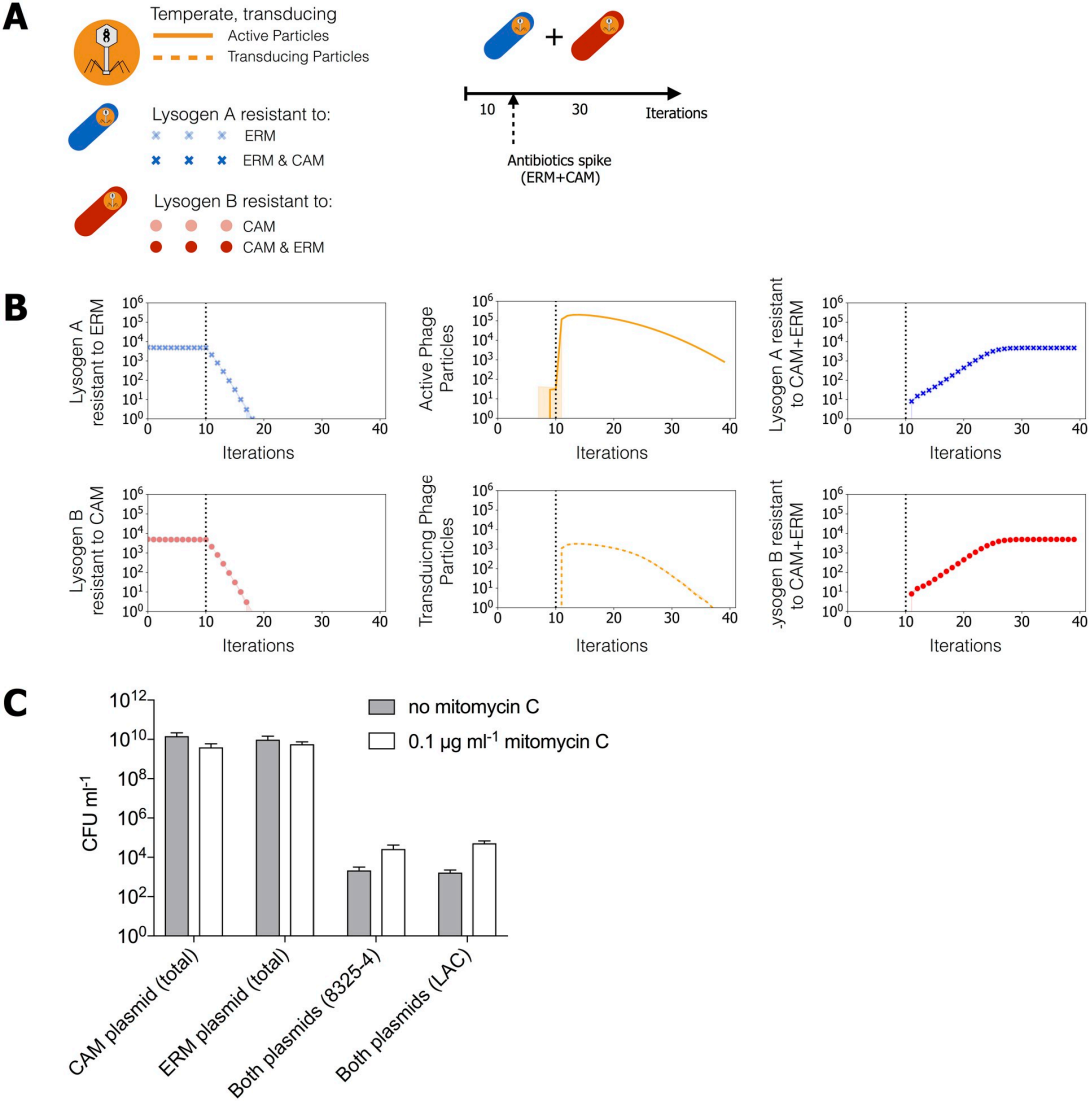
Gene-shuffling between genetically different strains lysogenized by the same phage. **(A)** The experiment includes two lysogens carrying the same generalized transducing temperate phage, but with different antibiotic resistance genes in their chromosome (lysogen A resistant to erythromycin, lysogen B resistant to choloramphenicol). (**B**) Population dynamics of bacteria and phage when bacterial strains are subjected to a cocktail of two antibiotics (ERM+CAM) at iteration 10. Bacteria resistant to one antibiotic only are shown in faded symbols (crosses or circles, first column), with full colored symbols indicating bacteria resistant to both antibiotics (third column). Phages particles are shown in orange (second column), with full lines for active particles and dashed lines for transducing particles. Lines correspond to the median of 100 different simulations with similar parameters, and the shaded areas correspond to a confidence interval of 95%. (**C**) Two strains of 8325–4 (AA001) and USA300 (AA002) background, respectively, harboring different plasmids with unique antibiotic resistance markers were mixed 1:1 to OD600 = 0.01. After incubation over-night, number of cells from the two different strain backgrounds carrying one or both plasmid-encoded antibiotic resistance markers were determined.

We first addressed this issue using modelling. We simulated a mixed bacteria culture, with two bacterial strains, where both are lysogens but each carry a different antibiotic resistance gene (Fig 6A). In this model we assumed that transfer occurs by generalized transduction with the transferred marker being present either on a plasmid or distantly located from the prophage integration site. Following an initial growth period, the two antibiotics were introduced in the environment. We observed that the lysogens were predicted to survive the dual antibiotic exposure (Fig 6B). A control simulation shows as expected that, in the absence of prophages, the entire population became extinct, since each of the strains resisted one but not both of the drugs (S4A Fig). Analysis of the genomes of the individuals from the simulations in Fig 6B showed that they were predicted to have acquired the missing antibiotic resistance gene through transducing particles originating from the other strain (S4D Fig), effectively exchanging the resistance traits. Importantly, these double resistant variants emerged even in the absence of antibiotics (i.e., in the absence of selection and antibiotic-associated induction), when spontaneous prophage induction was sufficiently high (S4B and S4C Fig).

To experimentally confirm the theoretical observations we grew, in a 1:1 ratio, two strains of *S*. *aureus* both lysogens for ϕ11. *S*. *aureus* AA001 is a derivative of 8325–4 carrying pRMC2 expressing chloramphenicol resistance and AA002 is a USA300-LAC containing the LAC-p03 plasmid encoding erythromycin resistance (Fig 6C). Again, as both resistance markers are located on plasmids we monitor generalized transduction. After overnight growth the abundance of cells resistant to either chloramphenicol, erythromycin or both antibiotics were determined on selective agar plates. Because of differences in pigmentation and hemolysis-phenotype between the 8325–4 and USA300 LAC backgrounds (S5 Fig) we were able to assign the resistance profile to the strain background. As seen in Fig 6C each of the two initial strains reached approximately 1x10^10^ CFU ml^-1^ but importantly, approximately 1x10^3^ CFU ml^-1^ of each of the strains had become resistant to both antibiotics indicating that both strains, in addition to their own plasmid, had received the resistance gene from the co-cultured strain. The co-culture experiments with strains lacking ϕ11 did not lead to double resistant colonies excluding the possibility that the plasmids were transferred by conjugation or natural transformation. Furthermore, in the presence of a sublethal concentration of the DNA damaging agent, mitomycin C, we observed both increased release of phage from the co-cultured strains (S6 Fig) and a 10-30-fold increase in transduction frequency (Fig 6C) supporting that DNA transfer occurs by generalized transduction.

In sum, both the theoretical and the experimental results support the conclusion that generalized transduction is a powerful mechanism of DNA transfer between strains, allowing the emergence of single and even double resistant variants. Hence, generalized transduction allows the phage to gain access to the pan-genome of the infected bacteria and generate novel genetic strain variants, which can increase the chances of survival for both the phage and its host in changing environments.

## Discussion

Generalized transduction has long been used as a tool for genetic engineering [22] and, at the same time, is considered a major driver of bacterial evolution [9,14,16,34–36]. Yet, we know relatively little of the process under natural conditions. Here we have examined the impact of generalized transduction on the interactions between temperate, transducing phages and two important bacterial pathogens, namely the Gram-positive *S*. *aureus* and the Gram-negative *S*. *enterica* Typhimurium. We find that with the examined pathogens and conditions, transduction and lysogeny contribute synergistically to the survival of transducing phages and their hosts. This is because transductants that are lysogens receive the genetic information without enduring mortality by the surrounding phage virions, leading to rapid spread of a trait when there is selection for it. In the rare cases where transductants are not becoming lysogens we anticipate that the cells have become resistant to phage attack by another mechanism. Once lysogens have established, they can exchange genetic material via transducing particles without the risk of being killed by the phage. In our experiments we monitored transfer of plasmid-encoded antibiotic resistance markers suggesting that transfer occurs by generalized rather than lateral transduction, a process which just recently was demonstrated to enable lysogens to transfer chromosomal markers positioned downstream of the phage integration site at very high frequencies [15]. We predict that if we were to monitor such markers even greater transfer frequencies would be observed.

The benefits of generalized transduction to the phage questions the idea that it is just an error in phage packaging. The potential for natural selection on phage to set the rate of transduction is further supported by the existence of genetic variability in these rates. In *Salmonella* phage P22 [37] and *E*. *coli* phage P1 [38], mutants have been found with either increased or decreased transducing ability. In P22, this was shown to depend on point mutations in the small terminase, the protein that recognizes the pseudo-*pac*-site and initiates packaging during transduction [39]. Moreover, a wide range of transduction efficiencies is observed among naturally occurring phages of both Gram-positive and Gram-negative bacteria with many being unable to transduce [40–42]. On the host side, bacteria benefit from the immunity, prophages confer towards related phages [43] and from prophage encoded products such as virulence factors [16] [44]. Once established, the transducing prophages may acquire genes from other cells through autotransduction [9]. Combined with the results provided here where phage survival is enabled through lysogeny and acquisition of traits such as antibiotic resistance, transduction appears as a cooperative strategy undertaken by some phages to ensure survival of the host. The computational model used here allowed a mechanistic exploration of the different parameters involved in these phage-bacteria interactions, and a qualitative prediction of experimental conditions that are not easily captured by traditional modelling approaches [45]. The model allowed an exploration of conditions that are not experimentally feasible, such as a fine modification of transduction rates. Furthermore, the analysis of the simulations performed with eVIVALDI has identified generalized transduction and lysogeny as the key promotors of both bacterial and phage survival, since variation in other parameters have, by themselves, a limited effect on the outcome of the simulations.

The apparent benefits of generalized transduction, and the absence of prophages from around half of the bacterial genomes [1], prompts the question of why not all bacteria are lysogenized with transducing phages and why not all temperate phages are tranducing. First, transduction may be considered costly as it is associated with fewer infectious particles due to packaging of bacterial rather than phage DNA and with fewer viable bacteria due to cell lysis. This cost could explain why non-generalized transducing phages exist in nature. Secondly, bacteria protect themselves against phages by various resistance mechanisms such as abortive infection, restriction-modification systems and CRISPR [46,47]. Defence systems based on the prevention of phage absorption will prevent the entry of DNA from transducing particles and restriction-modification systems will block transfer if the DNA originates from a strain lacking the system. In those cases, acquisition of new traits may take place by other types of mobile genetic elements such as conjugative plasmids or by natural transformation. CRISPR-Cas systems can recognize specific sequences of phage DNA and restrict phage entry into bacterial cells [48]. In this case, generalized transduction may still occur and may even be promoted by the CRISPR-Cas activity as it helps transductants to survive by blocking phage infection [49]. For the bacteria investigated in the present study, lysogeny is highly common with essentially all clinical strains being lysogens [4,50]. CRISPR-Cas systems are only found in a subset of strains and exchange of phages and DNA frequently occurs within lineages formed by the barriers of restriction-modification systems [50]. In these bacteria, cooperation with temperate and potentially transducing phages may be a survival strategy that not only allows exchange of mobile genetic elements and resistance genes but potentially also shapes the evolution of the bacterial genome within lineages.

When we examined the bacterial content of a phage lysate from the staphylococcal phage ϕ11, we found that the amount of bacterial DNA carried in phage lysates supports the transduction of un-linked chromosomal and plasmid markers and that each millilitre of phage lysate will contain approximately 20,000 copies of the bacterial genome. We have previously seen that there is substantial spontaneous release of ϕ11 from a lysogenic strain and have proposed that this may be important for the acquisition of traits by auto-transduction whereby released phage propagate on susceptible bacteria in the surroundings and returning transducing particles provide resistance to for example antibiotics [9]. Phage particles released spontaneously from or by induction of lysogens are likely to carry even greater percentages of transducing particles as they may be released as part of lateral transduction [15]. These transducing particles will be able to exchange genetic information between cells carrying the same temperate phage as we show here indicating that lysogeny with a transducing phage enables genetic exchange either by general or lateral transduction. In conclusion, our data show that generalised transduction is a mutualistic trait that promotes the survival of phage and lysogen alike and importantly that temperate phages benefit from transduction. While the potential for evolutionary conflict between phage and host clearly still exists, the strongly overlapping interests of temperate phages and their hosts are expected to help stabilise their relationship, and may explain why lysogens are so common in species like *S*. *aureus* and *S*. Typhimurium.

## Materials and methods

### Bacterial strains, phages and growth conditions

Bacteria and phages used are listed in S2 Table. *S*. *aureus* strains were grown in Trypsic Soy Broth (Oxoid) with the addition of 10mM CaCl_2_ when phage propagation was desired. *Salmonella* Typhimurium and *E*. *coli* strains were grown in Luria-Bertani Broth (Sigma). Incubation was done in 15 ml disposable centrifuge tubes at 37 °C with shake (200rpm) unless otherwise noted. Erythromycin (10 μg ml^-1^), Kanamycin (30 μg ml^-1^), Chloramphenicol (10 μg ml^-1^) or Tetracycline (5 μg ml^-1^ or 20 μg ml^-1^), all purchased from Sigma, was added when appropriate.

### Phage propagation, enumeration and test for phage resistance

Phages were induced from lysogenic strains by mitomycin C (2 μg ml^-1^, Sigma) at OD_600_ of 0.3 at which time the cultures were incubated at 32°C with 80 rpm shake overnight. The resulting lysate was sterile filtered and enumerated using the soft-agar overlay method. Plate lysates were prepared by harvesting soft agar plates with phages in SM buffer followed by sterile filtration. Phage resistance was examined by first spotting 10 μl of the given strain (overnight culture) as well as a susceptible indicator strain (for positive control) on an agar plate containing soft agar. After brief desiccation 5μl of an appropriate phage lysate allowing detection of 25–50 plaques on the indicator strain was spotted on top of the test strain and the indicator strain. Resistance was determined by the absence of plaques after overnight incubation on the test strain but not on the indicator strain.

### Construction of strains and phages

Plasmid and chromosomal markers were transferred between strains using standard phage transduction protocols [51]. To produce the virulent ϕ80α, allelic exchange was performed using derivatives of plasmid pMAD [52] carrying ΔcI, as described previously [53]. Plasmid pJP1686 (ΔcI) was constructed by cloning PCR products obtained with the following primers into vector pMAD: orf6phi80alpha-3mB (5’-CGCGGATCCTTTGCTTTGTTTAGAAGCATCG-3’), orf6phi80alpha-4c (5’-CTTCCGTTCAGACATAATTTG-3’), orf6phi80alpha-5m (5’-AAATTATGTCTGAACGGAAGAAGTATGATGATATCAAAGTCGC-3’) and orf6phi80alpha-6cE (5’-CCGGAATTCTTTCTCTTCCATCCCTCATCC-3’).

### Transduction and lysogenization experiments

Lysate from phage propagated on strains carrying a transferable marker was used to infect a recipient strain with a multiplicity of infection (MOI) of 1 at a cell density of OD_600_ = 0.01. Following overnight incubation, the number of cells (colony forming units, CFU) and transductants were determined by plating on non-selective and selective plates, respectively. The number of lysogens was determined for ϕ11-ERM by enumeration of erythromycin resistant colonies or, in the cases where no antibiotic marked phage was used, by inducing 96 transductants with mitomycin C (2 μg ml^-1^) and determine the presence of free phages on a suitable indicator strain. In time course infection experiments, ϕ11-ERM was propagated on a donor strain containing the non-conjugative plasmid, pRMC2. Using multiplicity of infection of 1, the cultures were infected at OD_600_ = 0.01 and grown for 16h in batch cultures and at regular intervals, 30 transductants selected on chloramphenicol were tested for erythromycin resistance. When antibiotic selection was applied in liquid cultures infected cultures were diluted 1000-fold in TSB containing 30μg ml^-1^ chloramphenicol and allowed to grow for 7h before another 1000-fold dilution in TSB containing chloramphenicol was applied. After overnight incubation the number of chloramphenicol-resistant transductants and CFU was determined in the cultures.

### Ratio of transducing particles to infective phage particles

qRT-PCR was used to determine the ratio of transducing particles to phage particles as previously described [29] including treatment of phage lysate with RNaseA and DNaseI at 1 and 5 ug ml^-1^, respectively. Briefly, control plasmid DNA was purified from a pUC18 vector containing the 4867-bp HindIII DNA fragment of bacteriophage ϕ53 (GenBank accession number, AF513856) designated p53D and a 139 bp product specific for all serogroup B phages [2] was amplified by the primers 53D -F (CGACAAAAGGCATTCAACAA) and 53D-R (ACGTTCAAAAATCGCTTGCT). The same primers were used for qRT-PCR reactions in reactions that contained 10ul of 2xSYBR green master, 500nM of each primer and 5ul of template DNA being either plasmid or phage DNA. For the standard curve, the numbers of control plasmid DNA molecules in reactions ranged from 3x10^7^ to 3x10^2^ in 10-fold fashion. The program of qRT-PCR is 10min of preincubation at 95°C followed by 45 cycles of amplification (95°C for 10s, 58°C for 10s and 72°C for 10s) and the final melting (95°C for 15s, 65°C for 60s and 97°C for 1s). According to the standard curve, which was made with the data of control plasmid, the copy number of infective phage particles was calculated. On the other hand, plasmid pRMC2 was used as standard to calculate the absolute copies of transduced plasmid DNA in the same lysate. The primers used in the second qRT-PCR was CAT-F (5´-TGGTTACAATAGCGACGGAGA-3´) and CAT-R (5’-TACAGGAGTCCAAATACCAGAGA-3´). The reactions were almost the same as the first ones except for primers and template. For the standard, the numbers of pRMC2 plasmid DNA molecules in reactions ranged from 3x10^6^ to 30 in a 10-fold fashion. The program was 10min of preincubation at 95°C followed by 45 cycles of amplification (95°C for 10s, 62°C for 10s and 72°C for 10s) and the final melting (95°C for 15s, 65°C for 60s and 97°C for 1s). According to the standard curve of pRMC2 plasmid, the copy number of transducing particles was calculated. Ratio of transducing particles to infective phage particles was calculated with the numbers of two types of particles.

### Detection of transduced DNA in phage lysates by PCR and sequencing

Phage lysate was treated with RNaseA (Thermo Fisher) and DNaseI (Thermo Fisher) at 1 and 5 ug ml^-1^, respectively to remove bacterial DNA and RNA as previously described [29]. DNA was prepared from 1 ml of the lysates used in the infection experiments (Table 1) using the MAGattract HMW DNA kit (Qiagen) according to the manufacturers’ instructions. PCR was performed on the isolated phage-lysate DNA with pRMC2 plasmid DNA as positive control and untreated phage lysate as negative control for free pRMC2 DNA in the lysate. Positive PCR reactions were confirmed to be originating from pRMC2 by sequencing. Primers pRMC2_fw (5’-GCGACGGAGAGTTAGGTTATTGGG-3’) and pRMC2_rev (5’-ACCTTCTTCAACTAACGGGGCAGGT-3’) were used for PCR. Primer pRMC2_fw was also used for sequencing.

### Individual-based model of bacteria-phage interactions

Simulations were performed based on the model described in https://doi.org/10.1101/291328. Both bacterial cells and phage particles are independent individuals on an environment represented as a two-dimensional grid with Moore neighbourhood (the 8 connected grid spaces of each location, for a Moore distance of one). The environment is simulated as well-mixed, meaning that positions of bacteria and phage are randomized at each iteration. Bacteria are simulated either with or without a gene conferring the ability to survive antibiotic exposure, and this trait cannot be evolved due to genomic mutation, it has to be acquired by transduction. Both bacterial strains have a similar growth rate, but the gene that provides resistance to antibiotics carries a cost. Each location in the environment can hold a single bacterial cell and several phage cells. Free space is the bacterial resource to be consumed, and it is freed whenever bacteria die. Bacterial death can be intrinsic (e.g., of old age) or explicit (e.g., exposure to antibiotics or lysed by phage). When a free space is available, the neighbouring bacteria compete for reproduction. The outcome of the competition is chosen through a roulette wheel method that accounts for the fitness of each bacterium. The successful bacterium generates an offspring into the free space. Phages have different lifestyles (temperate or virulent) and can become defective phage particles due to generalized transduction. The decision between a lytic or lysogenic cycle varies across the types of phages, and depends on the concentration of free phage particles in their proximity. The host range of phage is similar for all phages and is the same for both bacterial species. The burst size is also similar for all phage types. The superinfection exclusion rules amongst phages is parameterized according to the experimental setup simulated. The parameters used in the simulations are described in detail in S1 Text.

### Random Forest Analysis

The Random Forest Analysis (RFA) is based on simulations performed with the model, covering 5000 random combinations of parameters, with 20 simulated repeats per combination. The output of this cohort of simulations is grouped and resumed in response variables. This results in a large table with input parameters and response variables to which we add a column with 5000 rows of a random parameter (i.e., a choice of a number between 1 and 3). This parameter allows to assess the impact of random variables in the RFA. This table is used as input of the randomForest package in R (version 4.6.12), where the *randomForest* function is run with the parameters ntrees set to 10000. The relative importance of each parameter (the percentage increase in minimum squared error, %IncMSE) is assessed using the *importance* function from the same package. This function evaluates the effect of excluding each parameter on the ability of the RFA to predict the response variable (the percentage of simulations where phage survive, for our study). In the context of RFA, exclusion is performed by randomly assigning values to a parameter, rather than those used for the actual simulations. The predictive ability is compared between the original data and the permuted data (with the “excluded” parameter) and parameters that lead to high increases in prediction error (increased MSE) are deemed of high importance. A purposefully random parameter is also included in our analysis, to assess the impact of random noise in the quantification of the relative importance of the parameters. Non-important parameters have a small or null increase in MSE, similar to that of random noise. The parameters used in the simulations are described in detail in S1 Text and S3 Table.

## Supporting information

S1 Table qPCR determination of bacterial content in phage particles. (DOCX)

S2 Table Bacterial strains and phages. (DOCX)

S3 Table Parameters investigated. (XLSX)

S1 Text (DOCX)

S1 Fig PCR detection of pRMC2 in virulent phage lysate propagated on donor strains carrying pRMC2. Product of PCR reactions performed with pRMC2 specific primers and DNA isolated from 80α-vir phage lysate (lane1), from Φsa012 phage lysate (lane 2), pRMC2 plasmid preparation (lane 3), 80α-vir phage lysate prior to DNA purification (lane 4) and from Φsa012 phage lysate prior to DNA purification (lane 5).
(DOCX)

S2 Fig Genomic composition of simulated populations shows the acquisition of prophages and antibiotic resistance. A sample of 5 genomes from the initial timepoint of the first phase (with antibiotic resistant bacteria) and the second phase (with the antibiotic sensitive bacteria), as well as after 50 iterations in the latter, for a typical simulation. Red symbols represent DNA corresponding to antibiotic resistant bacteria, whilst green symbols represent DNA corresponding to bacteria (initially) antiobiotic sensitive. Rectangles represent essential genes and dashes represent non-essential genes. Losangles represent genes capable of conferring antibiotic resistance (with each color representing a gene capable conferring resistance to a different antibiotic), where bright colors identify genes that actively confer resistance and dimmed colors representing genes that require a mutation to acquire active resistance. Elipses show DNA that is associated with prophages. If they carry original phage DNA (meaning, the phage is intact), they are shown as orange ellipses. If they carry bacterial DNA, ellipses have the color of the respective bacterial DNA (i.e., light red for antibiotic resistant bacteria, light green for bacteria initially antibiotic sensitive). Antibiotic resistance genes, in their active or inactive state, keep the same color when represented within phage carried DNA. Once in the host genome, an horizontally transferred active antibiotic resistance gene will confer resistance to the respective antibiotic, even in the presence of another, inactive, antibiotic resistance gene. Note that, in the second phase, both transducing particles containing DNA from antibiotic sensitive bacteria (light green ellipses) and from antibiotic resistant bacteria (light red ellipses) can be found within surviving bacteria, and transducing particles from antibiotic resistant bacteria often carries the active antibiotic resistant marker, the orange losangle). Moreover, all surviving bacteria are also lysogens, meaning that the intact phage DNA (two orange ellipses) can also be found in its genome. Thus, surviving bacteria need to have acquired the resistance gene by transduction and to have become lysogens.
(DOCX)

S3 Fig Effect of generalized transduction and lysogeny for phage survival. **A**-**B**) Each dot corresponds to a single parameter combination (5000 in total). Each simulation was repetead 20 times, and the fraction of simulations that show phage survival (number of phage particles in the environment or prophages within bacterial genomes) are shown in the y-axis. **A**) Outcome relative to different values of probability of generalized transduction explored (x-axis) when the probability of lysogeny was “high” (see parameters in section 3 of Supplementary text file S1). **B**) Outcome relative to different values of probability of generalized transduction explored (x-axis) when the probability of lysogeny was “Middle” (see parameters in section 3 of Supplementary text file S1). This shows the impact of the frequency of generalized of transduction as long as there is some level of lysogenization, in spite of the extensive variation of the remaining parameters in these simulations.
(DOCX)

S4 Fig Exchange of resistances can occur spontaneously under high intrinsic induction, but not without lysogens. Setup of the simulations is similar to the one in Fig 6A. A) Population dynamics with non-lysogenic bacteria subjected to antibiotic exposure. B and C) Population dynamics with lysogenic bacteria in the absence of antibiotics, with different parameterizations of the model’s induction function (alpha = 100000, kappa = 0.4, equivalent to spontaneous induction rate of 10^−5^, for B; alpha = 1000, kappa = 0.4, equivalent to spontaneous induction rate of 10^−3^, for C). Lines correspond to the median of 100 different simulations with similar parameters, and the shaded areas correspond to a confidence interval of 95%. E) sample of the genomic composition of populations in Fig 6B at the beginning (t = 1) and sometime after antibiotic exposure (t = 18) of a typical simulation. Symbols are as explained in S2 Fig. CAM resistance genes are shown in orange (bright orange for resistant phenotype, dimmed orange for sensitive phenotype), whilst ERM resistance genes are shown in pink (bright pink for resistant phenotype, dimmed pink for sensitive phenotype).
(DOCX)

S5 Fig Colony appearance. Mixed culture of AA001 and AA002 grown on TSA (A) or TSA supplemented with 5% blood (B). AA001 (8325–4 background) appears white and non-hemolytic whereas AA002 (LAC background) appears yellow and hemolysis-positive.
(DOCX)

S6 Fig Phage release. Spontaneous and mitomycin C induced phage release from 8325-4-ϕ11 (AA001; p<0.05, Student t-test) and LAC- ϕ11 (AA002) (p<0.01, Student t-test) monocultures as well as mixed cultures of the two strains.
(DOCX)
