# Supplementary material for: Bacteriophages benefit from generalized transduction
Source: PLoS Pathog. 2019 Jul 5;15(7):e1007888. doi: 10.1371/journal.ppat.1007888 (PMC6636781; doi:10.1371/journal.ppat.1007888)
Supplement: S1 Table — (DOCX) [file ppat.1007888.s001.docx]

**Supplementary Table 1.**

| **Target DNA** | **Plasmid number** | **Cq value** |
| --- | --- | --- |
| 53D | 3x10^6^ | 15.48 |
| 53D | 3x10^5^ | 19.00 |
| 53D | 3x10^4^ | 22.20 |
| 53D | 3x10^3^ | 25.59 |
| 53D | 3x10^2^ | 29.50 |
| Phage lysat |  | 17.6 |
|  |  |  |
| pRMC2 | 3x10^6^ | 14.12 |
| pRMC2 | 3x10^5^ | 17.46 |
| pRMC2 | 3x10^4^ | 21.03 |
| pRMC2 | 3x10^3^ | 24.15 |
| pRMC2 | 3x10^2^ | 27.18 |
| Phage lysat |  | 19.7 |
