# Supplementary material for: Bacteriophages benefit from generalized transduction
Source: PLoS Pathog. 2019 Jul 5;15(7):e1007888. doi: 10.1371/journal.ppat.1007888 (PMC6636781; doi:10.1371/journal.ppat.1007888)
Supplement: S2 Table — (DOCX) [file ppat.1007888.s002.docx]

**Supplementary Table 2. Bacterial strains, plasmids and phages used**

| **Bacterial strains** | **Relevant characteristics** | **Reference** |
| --- | --- | --- |
| 8325-4 (RN450) | NCTC8325 cured of Φ11, Φ12, and Φ13 | [1] |
| RN4220 | Restriction-defective derivative of RN450 | [2] |
| JH930 | *S. aureus* 8325-4 carrying pRMC2, Cm^R^ | [3] |
| JH1064 | JH930, *agr::tet* (obtained by transduction from KT202), Tc^R^, Cm^R^ | This study |
| AA002 | USA300 LAC carrying LAC-p03 with φ11, Em^R^ | This study |
| AA001 | JH930 with φ11, Cm^R^ | This study |
| KT202 | TetM-cassette inserted in *agr* operon | [4] |
| JP13690 | *Salmonella enterica* serovar Typhimurium LT2 | [5] |
| JP13812 | *Salmonella enterica* serovar Typhimurium LT2, P22, pET28a | This study |
| JP14365 | JP13812 *prp*R::TetA, Tc^R^ | This study |
| JP9276 | *Escherichia coli* C600 | [6] |
| JP12712 | *Escherichia coli* C600, lambda, pET28a, Km^R^ | This study |
| JP8993 | DH5α pJP1686 (∆cI), Amp^R^ | This study |
| **Phages** |  |  |
| Φ11 | *S. aureus* temperate, pac-type, transducing phage | [7] |
| Φ11-ERM | Φ11 marked with erm-cassette, Em^R^ | [8] |
| Φ52A | *S. aureus* temperate, pac-type, transducing phage | [9] |
| Φ53 | *S. aureus* temperate, pac-type, transducing phage | [9] |
| 80α | *S. aureus* temperate, pac-type, transducing phage | [9] |
| 80α-vir | Φ80a, deletion of cI repressor, strictly lytic, non-transducing | This study |
| Φ12 | *S. aureus* temperate, cos-type, non-transducing | [7] |
| ΦSa012 | *S. aureus* naturally lytic phage, non-transducing | [10] |
| P22 | *Salmonella* temperate, pac-type, transducing | [11] |
| Lambda | *E. coli* temperate, cos-type, non-transducing | [12] |
| **Plasmids** |  |  |
| pRMC2 | Tetracycline-inducible expression vector | [13] |
| pET28a | A commercial vector with encoding kanamycin resistance | (Novagen) |
| pJP1686 (∆cI) | pMAD ΔcI 80 | This study |
| p53D | pUC18 vector containing 4867-bp HindIII DNA fragment of bacteriophage φ53 | [14] |

**References, Supplementary information**

1. Novick R. Properties of a cryptic high-frequency transducing phage in *Staphylococcus aureus*. Virology. 1967;33: 155–166.

2. Kreiswirth BN, Lofdahl S, Betley MJ, O'Reilly M, Schlievert PM, Bergdoll MS, et al. The toxic shock syndrome exotoxin structural gene is not detectably transmitted by a prophage. Nature. 1983;305: 709–712.

3. Haaber J, Leisner JORJ, Cohn MT, Catalan-Moreno A, Nielsen JB, Westh H, et al. Bacterial viruses enable their host to acquire antibiotic resistance genes from neighbouring cells. Nat Commun; 2016;7: 1–8. doi:10.1038/ncomms13333

4. Tegmark K, Karlsson A, Arvidson S. Identification and characterization of SarH1, a new global regulator of virulence gene expression in *Staphylococcus aureus*. Mol Microbiol. 2000;37: 398–409.

5. McClelland M, Sanderson KE, Spieth J, Clifton SW, Latreille P, Courtney L, et al. Complete genome sequence of *Salmonella enterica* serovar Typhimurium LT2. Nature; 2001;413: 852–856. doi:10.1038/35101614

6. Nakamura K, Takahashi K, Watanabe S. Myosin and actin from *Escherichia coli* K12 C600. J Biochem. 1978;84: 1453–1458.

7. Iandolo JJ, Worrell V, Groicher KH, Qian Y, Tian R, Kenton S, et al. Comparative analysis of the genomes of the temperate bacteriophages phi 11, phi 12 and phi 13 of *Staphylococcus aureus* 8325. Gene. 2002;289: 109–118.

8. Quiles-Puchalt N, Martinez-Rubio R, Ram G, Lasa I, Penades JR. Unravelling bacteriophage phi11 requirements for packaging and transfer of mobile genetic elements in *Staphylococcus aureus*. Mol Microbiol. 2014;91: 423–437. doi:10.1111/mmi.12445

9. Dowell CE, Rosenblum ED. Serology and Transduction in Staphylococcal Phage. J Bacteriol. 1962;84: 1071–1075.

10. Synnott AJ, Kuang Y, Kurimoto M, Yamamichi K, Iwano H, Tanji Y. Isolation from Sewage Influent and Characterization of Novel *Staphylococcus aureus* Bacteriophages with Wide Host Ranges and Potent Lytic Capabilities. Appl Environ Microbiol. 2009;75: 4483–4490. doi:10.1128/AEM.02641-08

11. Zinder ND, Lederberg J. Genetic exchange in *Salmonella*. J Bacteriol. 1952;64: 679–699.

12. LEDERBERG EM, Lederberg J. Genetic Studies of Lysogenicity in *Escherichia-Coli*. Genetics; 1953;38: 51–64.

13. Corrigan RM, Foster TJ. An improved tetracycline-inducible expression vector for *Staphylococcus aureus*. Plasmid. 2009;61: 126–129

14. Varga M, Kuntová L, Pantůček R, Mašlaňová I, Růžičková V, Doškař J. Efficient transfer of antibiotic resistance plasmids by transduction within methicillin-resistant *Staphylococcus aureus* USA300 clone. FEMS Microbiol Lett. 2012;332: 146–152. doi:10.1111/j.1574-6968.2012.02589.x
