# Supplementary material for: Bacteriophages benefit from generalized transduction
Source: PLoS Pathog. 2019 Jul 5;15(7):e1007888. doi: 10.1371/journal.ppat.1007888 (PMC6636781; doi:10.1371/journal.ppat.1007888)
Supplement: S1 Text — (DOCX) [file ppat.1007888.s004.docx]

Supplementary Text S1

[1. The ODD (Overview, Design concepts, and Details) Protocol of the eVIVALDI Model (version 9.2) 2](#_Toc535949)

[1.1 Overview 2](#_Toc535950)

[1.1.1 Purpose 2](#_Toc535951)

[1.1.2 Entities, State Variables and Scales 2](#_Toc535952)

[1.1.3 Process Overview and Scheduling 4](#_Toc535953)

[1.2 Design Concepts 6](#_Toc535954)

[1.2.1 Basic Principles 6](#_Toc535955)

[1.2.2 Emergence 7](#_Toc535956)

[1.2.3 Adaptation 7](#_Toc535957)

[1.2.4 Objectives 7](#_Toc535958)

[1.2.5 Learning 8](#_Toc535959)

[1.2.6 Sensing 8](#_Toc535960)

[1.2.7 Interaction 8](#_Toc535961)

[1.2.8 Stochasticity 8](#_Toc535962)

[1.2.9 Collectives 10](#_Toc535963)

[1.2.10 Observation 11](#_Toc535964)

[1.3 Details 11](#_Toc535965)

[1.3.1 Initialization 11](#_Toc535966)

[1.3.2 Input Data 12](#_Toc535967)

[1.3.3 Submodels 12](#_Toc535968)

[1.3.3.1 Bacterial Death 12](#_Toc535969)

[1.3.3.2 3.3.2 Prophage Induction 13](#_Toc535970)

[1.3.3.3 Phage Transduction 14](#_Toc535971)

[1.3.3.4 Phage Mutation 15](#_Toc535972)

[1.3.3.5 Phage Infection 16](#_Toc535973)

[1.3.3.6 Phage Lysis-Lysogeny Decision 17](#_Toc535974)

[1.3.3.7 Bacterial Reproduction 18](#_Toc535975)

[1.3.3.8 Bacterial Mutation 20](#_Toc535976)

[1.3.3.9 Environmental Degradation 21](#_Toc535977)

[1.3.3.10 3.3.10 Environmental Diffusion 23](#_Toc535978)

[1.3.3.11 Antibiotic placement in spatially structured environments 23](#_Toc535979)

[1.4 REFERENCES 23](#_Toc535980)

[2 Parameters used in the main analyses 26](#_Toc535981)

[3 Parameters used in the Random forest analysis 28](#_Toc535982)

# The ODD (Overview, Design concepts, and Details) Protocol of the eVIVALDI Model (version 9.2)

## Overview

### Purpose

The purpose of the model is to explore the role of bacteriophages in the dynamics of microbial communities, given both the role of phage as predatory agents and drivers of horizontal gene transfer between bacteria. The model aims to integrate and study the different mechanisms of predation and transfer (and their interactions) in representing real world scenarios of interactions between bacteria and phage. Additionally, the model’s purpose in its current state is also to provide a platform to incrementally add other genomic, ecological and evolutionary mechanisms to study microbial evolution.

### Entities, State Variables and Scales

The model has 7 hierarchical levels: **genes,** **genomes**, **bacteriophages**, **bacteria,** **locations,** **populations** and **environment**. Genomes are comprised of genes and are properties of the two types of agents in the model, bacteria and bacteriophages. Both bacteria and bacteriophage have dynamic genomes, due to the mechanisms of mutation and horizontal gene transfer. Bacteria and bacteriophages exist in a two dimensional environment, where each location (an (x,y) position) can be vacant or occupied by a single bacterium and none to multiple bacteriophage. A free location provides an opportunity for bacteria to reproduce, and represents a form of resource to be competed for.

Below we describe the variables of each hierarchical level, except for the populations, which have no explicit variables.

**Genes** are identified by their:

- Identifier character (A-Z), which identifies the species to which the gene belongs to in the beginning of the simulation.
- Size (in number of characters, a proxy for base pairs).
- Gene type (Coreand/or Mobile).
- Ability to confer antibiotic resistance. If a gene has this ability, an additional phenotype state is included (“Active” or “Inactive”).

**Genomes** are identified by their:

- Ordered list of genes. The initial genome size of bacteria is defined by a global parameter that is common to all species (**Number of genes**).

**Bacteriophages** are identified by their:

- Phage species name.
- DNA identifier character.
- Lifestyle (temperate, virulent or defective).
- Genome, which excludes the essential phage genes (assumed to be part of the phage). The explicit genome encompasses only a cargo that is composed of either non-essential genes or genes acquired from other bacteria.
- **Maximum cargo size**, the number of genes above which the phage becomes defective, also how many genes can be transmitted by generalized transduction.
- **Attachment morphology**, an internal number that identifies different attachments to bacterial hosts (common to a given species of phages, can be modified, through mutation, by individual phages).
- **Burst size**, identifies how many offspring virions are created from lysing a bacterium.
- **Lysogeny function**, defining the phage decision to integrate into the host genome (common to a given species of phages).
- **Induction function**, defining the phage decision to excise the host genome into which it is integrated (common to a given species of phages).
- **Probability of specialized transduction**, defining the probability of occurrence of this type of transduction during excision (common to a given species of phages).
- **Probability of generalized transduction**, defining the probability of occurrence of this type of transduction during virion packaging (common to a given species of phages).
- Current state (integrated within host, replicating within host, or free living in the environment).
- If integrated in the genome, the genomic position.
- Time, or iterations, passed in the current state (within or outside host).
- An adsorption efficiency towards every bacterial species defined initially (**Probability of adsorption**, specific between a given species of phages and a given species of bacteria).
- A superinfection protection efficiency towards every other phage defined initially (**Superinfection Exclusion Probability**, common to a given species of phages).

**Bacteria** are identified by their:

- Bacteria species name.
- DNA identifier character.
- Color.
- Genome composition and size (collection of genes and the state of the genes that are mutable).
- Growth rate.
- Location in the 2D grid.
- Resistance to antibiotics (redundant from genomic data).
- List of phage attachment morphologies to which the bacterium is resistant to.
- List of CRISPR cassettes.
- List of phages (currently infecting the bacterium or integrated in its genome as prophages).

**Populations** are the ensemble of individual bacteriophages and bacteria, and are identified by:

- Subgroups (*e.g.*, populations of the same species, or cells/phages with the same phenotype).
- Number of individuals of each type.

**Locations** are identified by their:

- 2 dimensional (x,y) position.
- A single bacterium (if the location is occupied by one).
- A number of bacteriophages (if any are located in this particular location).
- The local antibiotic concentration.

The **Environment** is identified by its:

- List of locations.
- Structure (well-mixed, spatially structured or semi-solid). A spatially structured environment fixes the individuals to their locations and the reproduction of bacteria into the Moore neighbourhood (defined as the current location and its 8 adjacent locations in a squared lattice) of distance 1. Abiotic elements (i.e., antibiotics and free phages) do not diffuse from their locations. A semi-solid structure expands the neighbouring Moore distance of reproduction to 3 and creates a diffusion factor of similar distance for the abiotic elements (i.e., antibiotics and free phage) in each location at each time step. In a well-mixed environment, the reproductive distance is kept at Moore distance 1 but the positions of each element in the environment (bacteria, bacteriophage, antibiotics) are randomized immediately after reproduction. (see section 1.3.3.7 in **Submodels**).
- Continuity (whether the environment is bounded at its borders or toroidal, i.e., the entire environment is a continuum).

### Process Overview and Scheduling

Time is modelled as a succession of discrete iterations at a microbial scale. It is focused on the lifestyle of bacteria, i.e., bacteria cannot replicate more than once per iteration. Iterations much shorter than the minimal doubling time of the fastest growing bacteria in the simulation provide a finer granularity in time (at the expense of computer time).

Bacteria in the simulation are processed in a random order that changes at the beginning of each iteration to avoid biases. If the environment is set as well-mixed, then the location of all bacterial cells is also randomized.

The lifecycle of phage includes calculating the probability of excision (if it is an integrated prophage) and/or burst (if the phage is in the lytic cycle). If excision or burst occurs, it is also at this point that transduction of bacterial genes by phage can occur (see section 1.3.3.1 in **Submodels**). Upon burst, the offspring of the infecting phage are released into the environment. Once in the environment, phage can immediately infect new bacterial cells, depending on infection efficiency, or it can take more than one iteration for this infection to occur (see section 1.3.3.5). Importantly, since this process is stochastic, infection is heterogeneous, with some phage infecting faster than others. Infection of temperate phages is followed by the lysis-lysogeny decision (see section 1.3.3.6), which defines if the phage integrates the host genome or enters the lytic cycle. Bacteriophage cannot infect and burst a bacterial cell in the same iteration, but prophages can excise and burst within the same iteration.

The death of the bacteria, due to phage predation, antibiotic exposure or stochastic death, leaves their current location vacant, and this resource (space) is free to be consumed by other bacteria (similar to, e.g., ^1,2^). Each iteration provides an opportunity for bacteria to reproduce, depending on the availability of these free resources and the growth rate of each bacterium. The speed of reproduction is defined by this growth rate and, due to stochasticity, bacteria with small growth rates can require multiple iterations before producing an offspring, even in the absence of competition. The stochasticity inherent to reproduction leads to an asynchronous reproduction of the bacteria as a population, since some cells can reproduce faster than others. Once a bacterium is selected to reproduce, its offspring will occupy the vacant location. During bacterial reproduction mutations can occur in certain adaptive traits of the offspring (e.g., resistance to antibiotics or to phage), but gene loss can also take place in non-core genes. At each iteration, the process of selection and reproduction occurs for every vacant location, but there is no requirement that every location is filled at each iteration, because a) each bacterial cell can only reproduce once per iteration (if they already reproduced they are unaccounted for in the competition for other locations); and b) no candidates might be suitable to occupy the vacant location, due to either no bacterial cells in the vicinity or very slow growth rate from all the candidate bacteria (see details of this reproductive step in section 1.3.3.7).

Finally, each iteration also leads to the update of the environment. This includes the degradation of the abiotic elements present in the environment (antibiotics) and free phage, according to the parameterization of the simulations (see section 1.3.3.8). Additionally, at each iteration cells and abiotic elements are either randomized if the environment is set as well-mixed, or the abiotic elements and phage are diffused locally if the environment is set as semi-solid. The flow chart of the operations at each iteration is represented below.


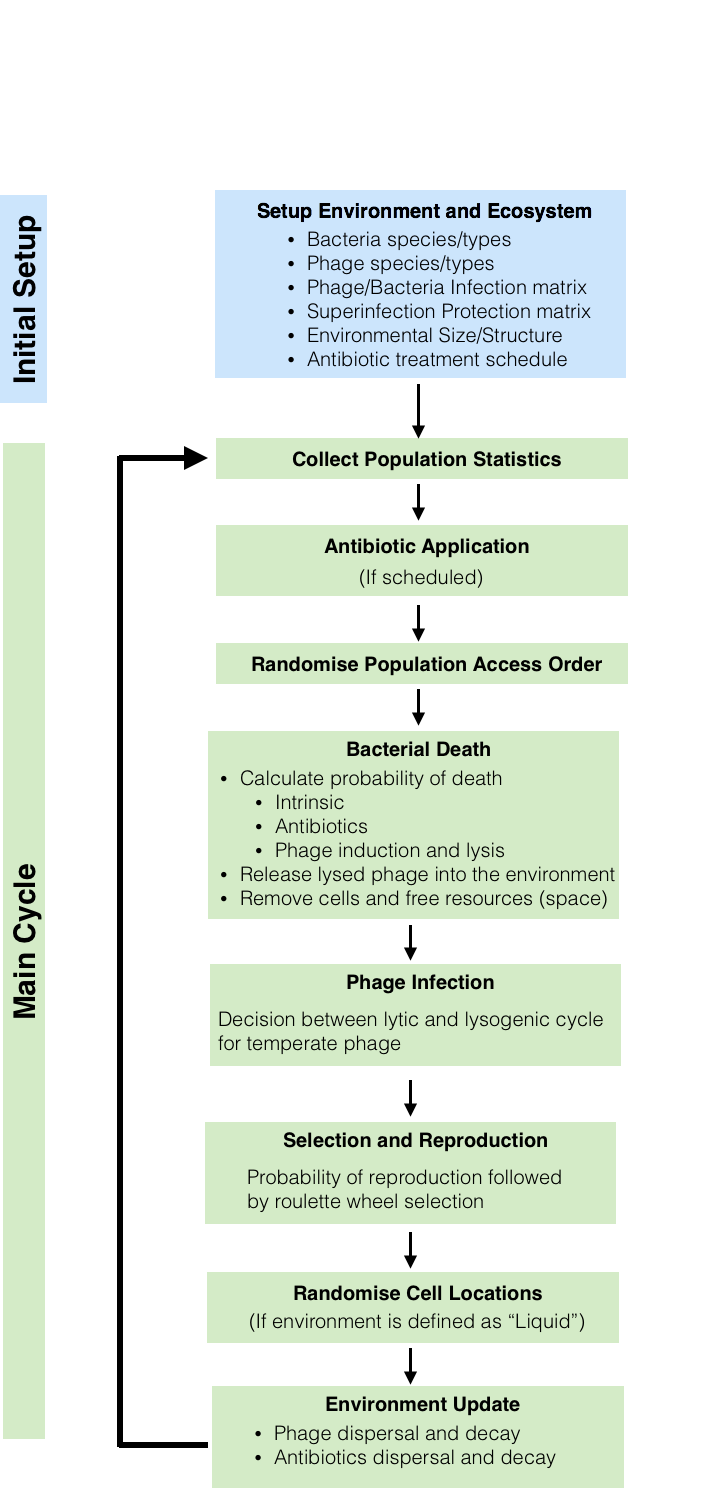


Figure 1.1. General flow-chart of the simulations.

## Design Concepts

### Basic Principles

The model aims to provide qualitative insights on the different mechanisms of interaction between bacteria and bacteriophages, in multi-species communities (of both bacteria and phage) under natural scenarios. The main principle of the model is the dual role of phage in both predating bacteria and serving as a mechanism of horizontal gene transfer. Thus, the main purpose of the model is to study how these two dynamics coexist and in which conditions one will prevail over the other. It is designed with a basis on current knowledge on these interactions, both at the molecular level (e.g., ^3^) and at the level of the population dynamics (e.g., ^4^). The mechanisms are implemented with the adequate level of detail in order to maintain the model general and computationally feasible.

### Emergence

Population and metapopulation dynamics emerge from the individual behaviour of bacteria and bacteriophage. Phage predation changes the composition of communities based on the phage infection host range (which is defined a priori), but the success of this infection can depend on whether bacteria are within reach of the phage or have become resistant to it. Moreover, in the case of temperate phage, the lysis-lysogeny decision is probabilistically based on the local viral concentration and thus emerges from the individual infections. The outcome of this decision (protection against further infections and use of prophage as weapons) has important, and emergent, effects in the composition of the communities.

The other major emergent aspect of the model is the phage-driven gene flow between bacteria. Phage predation (and bacterial acquisition of resistance to this predation) occurs concurrently with transduction of bacterial genes by phages. Hence, the traits that are transmitted between individual bacteria, the frequency of this transmission, and the way it impacts the survival of the bacteria (i.e., its selective advantage) emerge from the simulations.

### Adaptation

Both bacteria and phage can be subject to mutations in adaptive traits. For bacteria, there are two main selective pressures in the simulations: antibiotics and phage. Genes responsible for antibiotic resistance can switch between the “active” and “inactive” phenotype according to a mutation rate. A different mutation rate can add a particular phage attachment morphology to a list that defines to which phage an individual bacterium can resist infection. For phage, the main selective pressure is on their ability to infect bacterial hosts. During the lytic cycle, genomes of newly formed virions can be different from their progeny because they underwent mutation, at a certain rate, to change their attachment structure morphology, thus being able to escape bacterial defences for infection.

The growth rates of bacteria and the burst size of phage, as well as the diverse rates of mutation, transduction and infection, are immutable, and thus non-evolvable, during the simulations.

### Objectives

The major criteria of success of a bacterial species is its ability to avoid extinction in the face of the selective pressures, antibiotics and phage predation. When in competition with another species, success is also quantified by the frequency of one species over the other(s). A similar criterion can be applied to the phage.

### Learning

There are no learning mechanisms included in this version of the model.

### Sensing

Since the individuals have no access to global variables, such as the population size, both bacteria and phages regulate their behaviour according to the local information (i.e., from current or surrounding locations). Bacteria are affected by the presence of antibiotics in their current location which can modify the probability of death for a bacterium (depending on their phenotypic state regarding antibiotic resistance). Prophages are also indirectly affected by the local antibiotic concentration, as their induction can be mediated the SOS responses activated by, e.g., antibiotic stress ^5^. In the simulations, local antibiotic concentrations can increase the probability of prophage induction and excision from sensitive bacteria. The lysis-lysogeny decision is generally believed to favour lysogeny when phages are very abundant, even if there are very few mechanistic studies on the effect of the number of environmental phages on the decision of lysogeny. In phage lambda, lysogeny is favoured under high multiplicity of infection (MOI)^3,6^, which is directly proportional to the local phage concentration. In phi3T the probability of lysogeny is a direct function of the density of phages in the local environment by way of an analogue of a quorum-sensing mechanisms

^7,8^. In the model, after a successful infection, the lysis-lysogeny decision depends on the viral concentration in the surrounding locations (Moore distance of 1), which is used as a proxy for the MOI (we reasonably assume that if a sensitive bacterium is surrounded by a given number of phage, they will very likely co-infect the cell) in phages that work like phage lambda.

### Interaction

In the model, bacteria-bacteria interactions are always indirect, since no direct contact (from cell A to cell B) is explicit. These indirect interactions occur through competition for resources (space) and through the release of prophage in the environment that kills species B but not species A.

Direct phage-phage interactions occur in the lysis-lysogeny decision, which depends on the local phage concentration ^3^, and in superinfection exclusion (where prophages exclude infection by similar phages

^9,10^).

The infection (and death) of a bacterium cell by a phage is the main direct phage-bacteria interaction. However, the exchange of genes due to transduction, or the injection of transduced DNA by a defective phage are other types of interactions that can occur between these two entities.

### Stochasticity

Microbial communities and environments are highly heterogeneous ^11^, and the sources of variability are, in the majority of cases, not completely known. This means that some processes are intrinsically stochastic - including mutation-selection-drift - whereas others must be modelled as stochastic because we do not know the sources of variability^12^. The model represents these mechanisms by using stochastic or partly stochastic processes. The majority of these processes are parameterizable, and their frequency can be shaped by changing the respective parameters, studying its consequences for community composition and assessing the similitude with experimental observations.

The parameterizable stochastic processes are:

- Probability of bacterial reproduction (**growth rate** parameter, **resistance costs** parameters and roulette wheel selection process).
- Mutational process (**mutation rate** in the antibiotic resistance gene(s), rate of acquisition of a new morphology to the repertoire of resistances to phage and **probability of gene loss** in bacteria; **mutation rates in receptor or CRISPR sequence in phage**).
- Probability of infection by nearby phage (**adsorption probability** parameter, **infection distance** parameter).
- Probability of bacterial survival to infection by another phage (**superinfection exclusion** parameter).
- Probability of bacterial survival upon infection by a phage caused by CRISPR cassette acquisition (**CRISPR acquisition** parameter).
- Prophage inactivation probability(**inactivation probability** parameter, leads to defective prophage).
- Transduction probability, both specialized and generalized(defined by the parameters **probability of specialized transduction** and **probability of generalized transduction**. The former occurs per phage induction, the latter occurs per virion created during burst).
- Probability of a phage in the lytic pathway to burst(**burst probability** parameter).

There are also processes where the stochasticity of the mechanism is influenced by state variables. These are:

- Lysis-lysogeny decision(2 parameters define a logistic curve as a function of the local viral concentration, **lysogeny alpha** and **lysogeny kappa**, see section 1.3.3.6).
- Prophage induction decision(2 parameters define a logistic curve as a function of the local antibiotic concentration, **induction alpha** and **induction kappa**, see section 1.3.3.2).
- Probability of bacterial death and sensitivity to antibiotics (3 parameters define the death function of bacteria, which is a function of the concentration of antibiotics in the bacterium’s location, **death curve A**, **death curve B** and **death curve M**, see section 1.3.3.1).
- Probability of phage elimination(per phage, one parameter defines an exponential decay as a function of phage time outside any bacterial host, **phage decay lambda**, see section 1.3.3.9).
- Antibiotic decay(per location, one parameter per antibiotic defines an exponential decay as a function of iterations passed since the antibiotic was deployed in a specific location, **antibiotic decay lambda**, see section 1.3.3.9).

Some other processes are completely random. These are:

- The location of antibiotic resistance gene(s) during genome creation (one gene is selected at random from the entire bacterial genome for each antibiotic).
- The integration site of prophage (a position between genes is chosen at random from the bacterial genome).
- The bacterial DNA transduced during specialized transduction (one gene selected at random from nearby host DNA, where the maximum distance is parameterizable by **Maximum Genomic Distance for Specialized Transduction**).
- The bacterial DNA transferred during generalized transduction (selected at random without replacement from the host DNA, where the number of genes transduced are parameterizable per phage species (**Transduction Max Cargo**), but constant for all generalized transduction events).

Finally, some processes which are presently deterministic could become stochastic in future versions of the model:

- The distance used for reproduction and diffusion in semi-solid environments (fixed at Moore distance 3).
- The number of possible attachment morphologies that phage can express and bacteria can become resistant to (fixed by **possible receptors** parameter).
- The infection distance for phage to infect a bacterial cell (fixed by **infection distance** parameter, set as Moore distance).
- The number of phage offspring after burst (fixed by **burst size** parameter).
- The distance neighbourhood used for the viral concentration assessment in the lysis-lysogeny decision (fixed at Moore distance 1).
- The maximum cargo before a phage becomes defective, defines also how many genes are transduced during generalized transduction (fixed by the **Transduction max cargo** parameter).

In some simulations where the environment is structured instead of well-mixed, the deployment of antibiotics is non-homogeneous. This is a particular mechanism, as it involves operations that are completely random and others with fixed parameters. This is detailed in section 1.3.3.11 in the **Submodels** section. This (admittedly) complex mechanism was chosen in order to provide an adequate balance between stochasticity and heterogeneity in applying antibiotics to spatially structured environments.

### Collectives

Collective entities are emergent from the model from the groups of individual cells, but no individual decisions take these collectives into account. The collective entities resulting from the simulations have no state variables or properties, and are meant to evaluate dynamics occurring at the population level resulting from individual mechanisms. They can be assessed at different levels which are, from the highest to lowest level:

- Number of total bacteria or the number of total phage.
- Bacteria from one species or the phage from one species.
- Bacteria with a given phenotype (e.g., antibiotic resistance) or genomic characteristic (e.g., integrated prophage), or phage with a given genomic characteristic (e.g., carrying a transduced antibiotic resistance gene).
- Bacteria in a given cellular state (e.g., ongoing lysis by an infecting phage).

### Observation

The data used for observation of the model is collected mostly from the collective entities described above, regarding the population scale of the simulations. However, depending on the analysis, data can be collected at the level of the individual (e.g., the distribution of the number of phages in bacterial cells) or even at the level of the genomes of individual cells (e.g., the localization of integrated prophages in bacterial genomes). The frequency of data collection can be parameterizable, and it occurs at the beginning of an iteration. The subsequent analysis of these observations can then take into account either all the collected data, or limit the observations to a unique measure to mimic the output of an experimental setting (e.g., the number of bacteria and phage, or the ratio between two bacterial species).

## Details

### Initialization

Much of the initial state of the simulations can be defined by the experimenter. The random seed itself can also be input as a parameter, in order to achieve reproducible simulations. Initial bacteria and initial phage (if any) are located at random in the environment. The initial properties that can be parameterized are:

- The different bacterial species, and their initial numbers in the population. For each species:
  - Presence/absence of antibiotic resistance phenotypes.
  - Presence/absence of phage resistance phenotypes.
  - Presence/absence of integrated prophage.
- The different phage species, and their initial numbers (in the environment). For each species:
  - Lifestyle (temperate, virulent or defective).
- The type of environment (well-mixed, spatially structured or semi-solid) (**Environmental Structure**).
- The timing and amount of antibiotics applied (per location) (**Antibiotic Application Times** and **Antibiotic Concentration**), as well as the type of antibiotic deployment in spatially structured environments (Homogeneous or heterogeneous, defined in **Antibiotic Arrangement in Structured Environments**).
- The number of possible phage attachment morphologies, defining the phenotypic space of attachment morphology resistance by bacteria (**Number of possible phage receptors**).

Some other properties are fixed depending on the type of simulation required. These include:

- The initial cargo (genes) of phage, and whether it initially includes an antibiotic resistance gene.

### Input Data

The only type of input data used by the model is the time and period of application of antibiotics.

### Submodels

#### Bacterial Death

Biologically, death in bacterial cells can occur due to antibiotics, either by inhibiting reproduction (bacteriostatic drugs) or by performing lethal biochemical modifications (bactericidal drugs) ^13^. Exposure to antibiotics at low concentrations (below the minimum inhibitory concentration) can lead to the death of some cells, but it is compensated by the growth of the population as a whole ^14^. Death due to phage occurs by viral replication within the bacterial cell and the subsequent burst of the bacterium. The asymmetrical division of bacterial cells as also been argued to lead to a kind of aging that eventually disrupts the normal function of a bacterium, leading to its death ^15^. In the model, we simulate these 3 mechanisms as follows:

- Ageing is simulated by the cumulative probability of intrinsic death (when the antibiotic concentration is zero), which is independent at each iteration but accumulates as time passes. We assume that the individual probability of intrinsic death at each iteration also accounts for other random death events that are not explicitly modelled (e.g., starvation).
- We assume that only bactericidal antibiotics are used in the simulations, and thus contact with the drugs increases the probability of immediate death of a sensitive bacterium. If the bacterium is resistant to antibiotics, the latter have no impact in the probability of death of the former, regardless of their concentration.
- Bacteria infected by phage die when these burst from the cell. Once phage engage with the lytic cycle, the probability of burst (per iteration) is defined by the parameter **Burst Probability**.

The following equation determines the probability of bacterial death at a given iteration, by ageing (in its cumulative form), random death events or antibiotic exposure.

In the absence of antibiotics this results in a non-null minimal probability of death (by design). This function can produce a variety of mortality dynamics, including sigmoid functions as the ones of popular models in pharmacodynamics^16^.


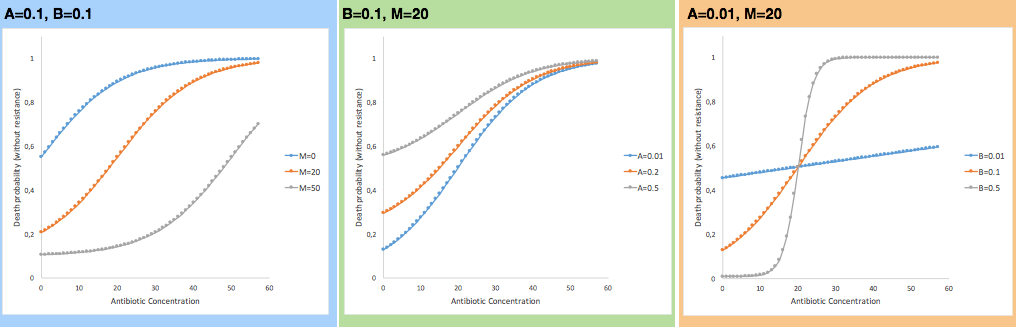


Figure 1.2**.** The different shapes of death curve function, where the probability of death at each iteration (y-axis) is given as a function of the antibiotic concentration (x-axis). When antibiotic concentration is zero, the function corresponds to the intrinsic death probability for each cell.

**AntibioticConcentration** is the amount of antibiotic present in the current location, and **Death Curve** **A, Death Curve B and Death Curve M** control the shape of the death function. These 3 parameters allow the experimenter to change the intrinsic death rate (i.e., the probability of death when AntibioticConcentration is zero), the rate of increase in the death probability as the antibiotic concentration increases, and the minimum inhibitory concentration of the drug. Note that by changing any of these parameters, bacteria with different intrinsic mortality rates (when antibiotics are absent) can be studied, allowing the study of this trait in the dynamics of populations^17^. The reasoning for the equation is based on a quasi-logistic function for the sensitivity to antibiotics, where low concentrations lead to a small or null increase in the death rate, then an intermediate region where there is heterogeneity in the death of the individual cells, and finally a region of high antibiotic concentration where bacterial death is certain. However, the three parameters allow the experimenter to explore more flexible functions of response to antibiotics. It can be fitted with experimental data, which shows different concentration-killing curves depending on the antibiotic or the bacteria ^18,19^. Finally, if death occurs due to phage burst, the offspring of the infecting phage (the number is defined by the **Burst Size** parameter) will remain in the position where the bacterium was.

#### Prophage Induction

The induction of a prophage and its entry into the lytic cycle can occur intrinsically, without apparent external stimulus, at low rates ^20^. The prophage can also be induced at much higher rates by external factors, e.g., DNA damage and/or antibiotic exposure ^21^. We simulate the induction of prophage according to the following equation, which is a function of the antibiotic concentration:

**AntibioticConcentration** is the antibiotic present in the current location

**Induction α and Induction κ** control the induction curve


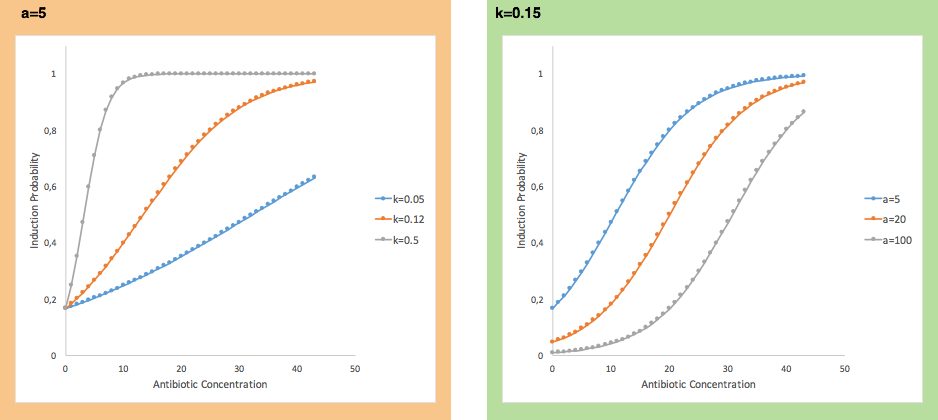


Figure 1.3**.** The different shapes of induction function, where the probability of induction at each iteration (y-axis) is given as a function of the antibiotic concentration (x-axis). When antibiotic concentration is zero, the function corresponds to the intrinsic induction probability for each cell.

The equation represents a logistic equation, as observed experimentally for the induction of phages with ultraviolet or mytomycin C, for instance ^22^. The two parameters in the equation control the intrinsic induction probability without antibiotics (parameter **Induction  **AntibioticConcentration=0) and the necessary antibiotic concentration to induce prophages at a high frequency (mostly defined by **Induction **). Induction of prophages can occur under minimum inhibitory concentration of antibiotics, if the induction function defines an increase in probability of induction under a concentration of antibiotics defined as sub-MIC in the death equation in 1.3.3.1.

#### Phage Transduction

Transduction by phage can occur by specialized transduction or generalized transduction ^23^. The first requires a lysogenic infection, where phage integrate into the bacterial genomes, and thus can occur only with temperate phage. It is characterized by an imprecise prophage excision upon induction of the element, where a piece of adjacent bacterial DNA is excised along with phage DNA. Generalized transduction can occur both with temperate and virulent phage, since it does not require phage integration. This type of transduction occurs during phage packaging, when a random segment of bacterial DNA, instead of phage DNA, is packaged into the viral capsid. The resulting phage is still able to infect, but since there are no viral genes, it is not able to replicate any longer.

In the model, the probability ofspecialized transduction is given by the parameter **Specialized Transduction Probability** and is associated with the phage species. Starting from the location of the prophage in the bacterial host genome, one gene will be selected from the list composed of the genes within the **Specialized Transduction Genomic Distance** parameter, in both directions of the genome (upstream and downstream). We assume that non-adjacent genes can be integrated, even though there are adjacent genes that are not. The integrated bacterial DNA can be a core gene, a non-coding gene, a resistance gene, or a piece of another mobile element residing in the genome. The chosen gene is added to the cargo of the phage at the end of its genome. If the total number of genes in the phage cargo after transduction (considering both the existing genes and the transduced ones) is above **Transduction Max Cargo Size** parameter, then the phage becomes defective. Since this occurs during excision, and before replication of the phage, all the virion offspring will carry the same transduced DNA.

**Generalised Transduction** is not necessarily associated with lysogeny (it can be undertaken by both temperate and virulent phage). Contrary to specialized transduction, where all virions will have the same transduced bacterial DNA, each virion produced might undergo generalised transduction independently, with **Generalised Transduction Probability**, which is defined for each phage species. Thus, if more than one generalized transduction event occurs during a single burst, the transducing virions are likely to carry different segments of bacterial DNA. The number of bacterial genes transduced is assumed to be always the same, but is specific for each phage species, and is defined by the **Transduction Max Cargo Size**parameter. This fits experimental data showing that the amount of packaged DNA varies on a narrow range and is a function of capsid size ^24^. The actual bacterial genes transduced are chosen randomly from the entire genome (and may include other mobile elements, including other genes contained in other phage cargo). The current cargo of the virion is completely replaced by this set of genes and the phage becomes defective.

**Defective phages** that are released into the environment (after either specialized or generalized transduction) are assumed to still be able to infect bacterial cells with the same efficiency of its non-defective variant, and integrate the transduced DNA into the bacterial genome. However, they cannot be induced, excise, or replicate within the bacteria.

#### Phage Mutation

Phages are known to have higher mutation rates than their hosts ^25^, and although this leads to a high load of deleterious mutations, it is also a key process in their ability to keep up with the evolution of bacterial defences to phage infection ^4^. Mutations in phage are known to allow them to escape bacterial defences based on changes on their tail fibers responsible for attaching to bacteria ^26^ or mutations in regions previously acquired by the bacteria as CRISPR cassettes ^27^.

In our simulations, we assume that beneficial, neutral or detrimental mutations can occur in phages, related with the ability of phage to escape bacterial defences and that and they take place during viral replication. First, virions can acquire mutations that change the morphology of their bacterial receptor. This does not represent a change in the host range of the phage, but instead allows them to successfully attach to their hosts (beneficial mutations). This occurs with a probability given by the parameter **Phage Receptor Mutation Probability**. The receptor morphology is characterized by a number, and the maximum number of receptors possible (i.e., the possible receptor phenotypes) is given the parameter **Possible Receptors**. Upon mutation, a random sample is performed of the values comprised between 0 and **Possible Receptors** (with the exception of the current receptor), and this new receptor morphology is assigned to the mutated phage.

From the point of view of the bacteria, we assume that CRISPR-based defences (see section 1.3.3.5 below) is based on the acquisition of a particular sequence present in each individual phage (a 10-character ASCII string). Virions can thus mutate this sequence, allowing them to infect bacteria that have previously acquired their (original) CRISPR sequence. This occurs with a probability given by the parameter **Phage CRISPR Mutation Probability**, and results in the random generation of a new 10-character string.

#### Phage Infection

The process of infection of a bacterium by a phage involves multiple processes, spanning the physical proximity between the two, the ability of phage to attach to the bacterial cell, and the injection of viral DNA into the bacterial host. Multiple mechanisms can prevent phage infection, including (but not limited to) avoiding contact with the phage ^28^, acquiring mutations that change the surface of the receptor responsible for phage attachment ^29^, or the presence of other phage in the cell prevents the injection and expression of invading viral DNA

^9,10^. Accordingly, the model attempts to reproduce infection as a multi-step process to allow the introduction of these mechanisms. The infection of a bacterial cell by a phage is first constrained by the proximity between the two entities. The minimum distance for infection is given by the parameter **Infection Distance**, which is measured as a Moore distance necessary for the contact between phage and bacteria. An **Infection Distance**of zero means that phage can only infect bacteria that are located in the same position as the phage. After this initial contact, a series of barriers have to be overcome:

- The probability of phage adsorption to the cell is given by the **Adsorption Probability** of the phage species in the bacterial cell’s species (i.e., the host range of the phage). Hence, some bacteria-phage interactions are much more likely to result in successful attachment at each iteration than others.
- Following attachment, the phage must surpass bacterial defences. They are, in the order they are checked:
  - Superinfection probability, represents the probability that a phage succeeds in infecting a bacterium that already harbors a phage. It is defined at the beginning of the simulations in the form of a matrix characterizing all pairwise interactions between the different types of phage. If more than one resident phage can provide superinfection protection, the success of an infection is assumed to be given by the one that can provide the highest amount of protection against the infecting phage.
  - Receptor defences, where bacteria must not have acquired mutations that prevent the phage infection (see section 1.3.3.8).
  - CRISPR defences, where bacteria may have acquired a CRISPR cassette that prevents phage infection. The acquisition of CRISPR cassettes can occur during an infection (if so, the infection fails and the phage is removed from the environment), and the bacterium adds the 10 character ASCII string representing the phage sequence to its CRISPR array.
- If infection failed due to receptor defenses or superinfection exclusion, the phage is kept in the environment, where it might attempt another infection to other bacteria (in the same iteration) or the same one (in the next iterations). On the other hand, bacterial CRISPR acquisition of an invading phage always removes the phage from the environment.
- If the bacterium is unable to use any of the defence mechanisms to prevent phage infection, then the phage is removed from the environment and it becomes part of the bacterial entity.

Once the infection has been successful, the counter of the number of iterations the phage has spent outside a host is reset to zero. The phage lifestyle defines what occurs next. Virulent phage will necessarily enter the lytic cycle, start replicating and prepare to burst (we assume that burst cannot occur in the same iteration as the infection). A temperate phage performs a lysis-lysogeny decision (see below). If the lytic pathway is chosen, then it becomes “Induced temperate” and it enters the lytic cycle, starts replicating and preparing to burst, like virulent phages do. If the lysogenic pathway is chosen, or if the incoming phage is defective, then the DNA carried by the viral particle integrates into the bacterial host genome, at a randomly chosen position.

#### Phage Lysis-Lysogeny Decision

The determinants of the lysis-lysogeny decision are not completely known, and are the subject of intensive research. The decision seems to involve some information on the amount of potentially competing phages, *e.g.*, through MOI or quorum-sensed based information on the number of phages in the environment ^6^

^8^. High values of these variables increase the probability of lysogeny (but the process remains stochastic). Exactly how this assessment is performed is most often not clear. Thus, this decision is simulated by taking into account the local viral concentration (i.e., number of phage within Moore distance 1) which is a proxy for the presumptive MOI in the host bacteria. It follows a probabilistic logistic equation, as observed experimentally ^3^. The equation chosen to model this decision allows to control the intrinsic rate of lysogeny (i.e., when there are no phage), and how fast the probability increases with an increasing number of surrounding phage. The equation is as follows:

**SurroundingPhage** is the number of phage within Moore distance 1

**α and κ** control the lysogeny curve


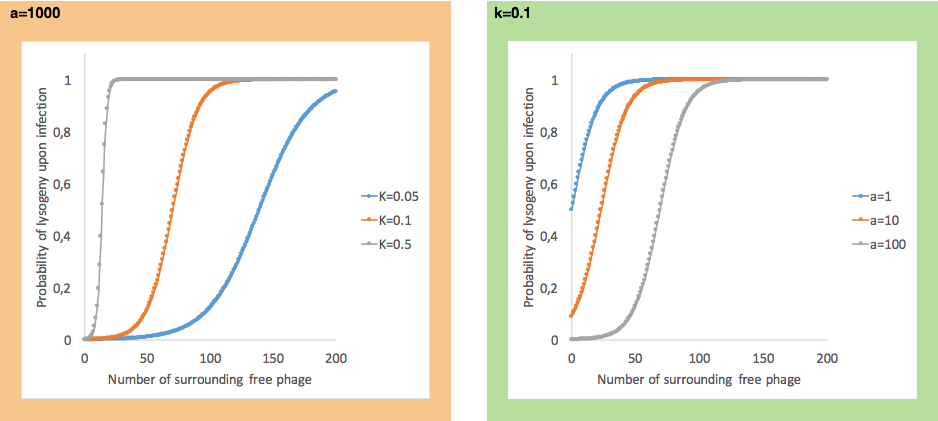


Figure 1.4**.** The different shapes of lysogeny function, where the probability of lysogeny at each iteration (y-axis) is given as a function of the number of phage in the Moore distance 1 (x-axis). When the number of phage is zero, the function corresponds to the intrinsic lysogeny probability for each phage.

#### Bacterial Reproduction

At each iteration, and for every vacant location, a competition between eligible bacteria defines which one (if any) will produce an offspring to occupy the location. There are four criteria for eligibility.

1) **Distance**. Bacteria must be less than a maximum distance from the vacant location (Moore distance of 1 for spatially structured or well-mixed and of 3 for semi-solid structure). The decision of keeping a distance of 1 for well-mixed environments rests on the assumption that cells do reproduce locally even in liquid environments, and it is the absence of structure that will quickly, and in a potential far-reaching distance, separate mother and daughter cells (which we reproduce by randomizing all cell positions at each iteration immediately after the reproductive cycle of the entire population). The Moore distance 3 for a semi-solid environment, on the other hand, represents a (potential) limited diffusion between the cells after reproduction.

2) **Growth rate**. The growth rate defined by the experimenter is corrected to account for the cost of resistances to antibiotics or phages (if relevant), resulting in a compound growth raterepresenting the fitness of the individual cell. These costs are also parameterizable by the experimenter, and affect the original growth rate in a multiplicative way:


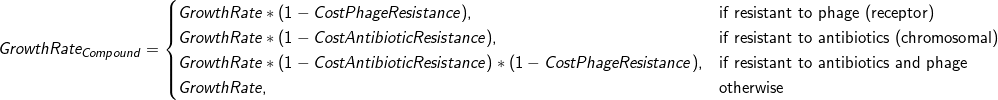


Based on this compound growth rate, cells can be discarded from entering the competition for reproduction in the current iteration. This decision is stochastic, meaning that cells with a low compound growth rate will enter the competitive round of reproduction less often. If the population is homogeneous (i.e., the growth rate is similar), this results in a slower growth of the population, since more iterations are required for the entire population to double in size. This decision depends solely in the growth rate of cells (and eventual costs), and has no account for other competitor bacteria.

3) **Previous reproduction**. The bacterium cannot have previously reproduced in the current iteration.

4) **Lysis**. The bacterium must not be infected by a phage that is producing lysis (virulent or induced temperate phage). If this is the case, the bacterium is ineligible to reproduce.

If, after these four conditions, no bacteria are eligible to reproduce and occupy the vacant space, this space remains vacant at least until the next iteration. If there are eligible bacteria, all of them will enter into direct competition for reproduction. Their compound growth rate is used in a roulette wheel process to choose the winning candidate. In the roulette wheel selection process, the competitive fitness (compound growth rate) of bacterium *i* is proportional to its probability of being selected to reproduce to the empty location, hence their probability of reproduction (higher for fitter cells) is influenced by their competitors. At every iteration, this process is repeated until all vacant locations are processed, but not necessarily occupied.

The model fundamental unit of time is the iteration. The classical concept of bacterial generation, as considered in population genetics, is not explicitly modelled here because it is not individual-centred, no evidence of frequent synchronization of replication in nature, and because there may be many different species with different growth rates in the simulations. Instead, the biological meaning of “generation” for a given species emerges from the simulations. If one wishes to translate the notion of iteration to that of generation one should consider that a growth rate of (e.g.) 0.5 (meaning 50% probability of replication in the current generation) implies that a cell with access to resources (space) and in the absence of competition divides, on average, every two time-steps (iterations). However, a generation in terms of the population is a compound factor of the effective growth rate and of the mortality rate of the individual cells that are the result of the amount of resources available, the presence of stressors (that increase mortality) and the population size (competition for resources). The ensemble of these computations across all individual cells will result in a population growth rate, and this (along with the time needed for a doubling of the number of bacteria) will change as the populations change in size and the availability of resources (space) changes. Without any stressors (i.e., antibiotics or phage predation), bacteria will divide until they reach the limit of resources (i.e., the number of available individual spaces. However, at this stationary demographic state, fluctuations can occur in the model due to death of existing cells, liberation of their resources (space), and their replacement by new births that occur when resources are made available. In the example shown in Figure1.5 (green line) below, 100 bacteria, of a species whose growth rate is 0.5, were inoculated at time 0. According to the classical calculation of the number of generations (2^n^ x B_0_ = B_final_, solving for n), it took ~13.3 generations to reach stable demography (until iteration 18), or 0.74 generations per iteration. For a slightly lower growth rate (red bacteria) 13.1 generations elapsed in the same number of iterations, whilst a higher mortality rate (blue line) show a lower number of generations (~10) even if the growth rate is similar to the ones in the bacteria from the green line. This shows that different cellular parameters (division and mortality) account not only for the fate of an individual cell, but also for the assessment of population-level dynamics such as the generation time.


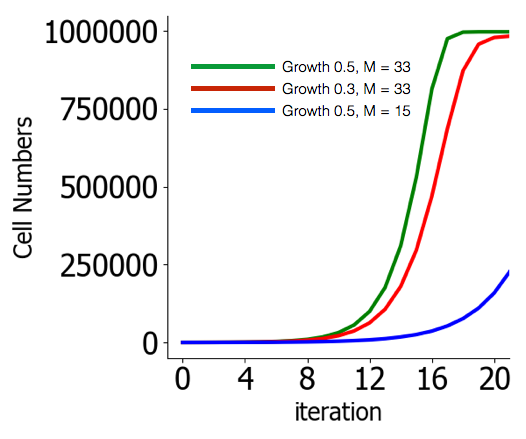


Figure 1.5**.** Typical growth curves obtained with the model, for bacteria with different parameters growing in well-mixed environments. At time 0, 100 cells of bacteria were inoculated and grown for 22 iterations. For these simulations the limiting resource, or the number of individual locations in the environment, is 10^6^. Green and blue lines show bacteria with similar growth rates but a higher mortality rate affects the bacteria represented by the blue lines. Red line shows bacteria with a mortality rate similar to green bacteria, but with a lower growth rate.

The previous observation is also associated with the concept of “iteration” (the atomic time-step of the model), whose biological meaning might not be immediately obvious. In essence, the experimenter can define the time equivalent of an iteration by changing the growth parameter of a bacterial species. For instance, if one considers *Escherichia coli*, which has been observed to divide (under optimal growth conditions) every 20 minutes, and if a growth rate of 0.5 corresponds to a division every (average) 2 iterations, then one iteration will correspond roughly to 10 minutes.

#### Bacterial Mutation

When reproducing, bacteria generate offspring that are identical copies of themselves, including the genome, the (eventual) phage, and CRISPR cassettes. However, some traits can undergo a mutational process to differ from the parent cell. These are:

- Antibiotic resistance genes, which can switch between the “inactive” and “active” state according to the (same) probability given by the parameter **ARG Mutation Probability**. This is assessed in a gene-by-gene fashion, where all putative resistance genes, be it on the original genome or on genes carried by phage, may mutate.
- List of phage receptors morphologies avoided by the bacterium, which can increase according to the probability given by the parameter **Resistance Phage Receptor Mutation Probability**. As for the mutations in the phage themselves, the maximum number of receptors possible (i.e., the possible receptor phenotypes) is given the parameter **Possible Receptors**. Upon mutation, a random sample is performed from the values comprised between 0 and **Possible Receptors** (with the exception of the receptors that are already in the list of the bacterium), and this new receptor is added to the list of resistances.
- Gene loss. Non-core or mobile elements can be deleted from the genome with probability (per gene) given by the parameter **Gene Loss Probability**.

#### Environmental Degradation

In the model, there are two elements subject to degradation through time: antibiotics and phage particles outside bacterial hosts.

Antibiotics are degraded with an exponential decay, assuming first-order kinetics following observations from the literature^30^, with parameter **Antibiotic Degradation Lambda** (independent for each type of antibiotic),and their concentration decreases along time following the equation:

**Concentration** is the antibiotics concentration in the current location

**t** is the current iteration

**λ** is the decay factor

Figure 1.6**.**  The antibiotic decay function, where the antibiotic concentration (y-axis) is decreased as function of time (x-axis). Different rates of decay are obtained with different lambda parameters.

Individual phage particles disappear along time also according to an exponential-decay function^31^. However, since there is no continuous degradation of the phage, this function gives the probability for a single phage to disappear from the environment according to the time that it has lived in the environment (i.e., outside a host). In terms of population size, this implies also a quasi-exponential decay (due to stochasticity). The probability of phage elimination from the environment was approximated by the equation:

**TimeOutsideHost** is the time the phage has spent in the environment

**λ** is the decay factor

Figure 1.7. The phages’ elimination (and phage population decay) function, where a single phage’s probability of being kept in the environment decreases as function of time it has spent outside a host (x-axis). Different rates of population decay are obtained with different lambda parameters.

#### Environmental Diffusion

Diffusion of phage particles and antibiotics occurs when the environment is set as well-mixed or semi-solid.

For well-mixed environments, the antibiotic concentrations of each location are randomly re-assorted to new locations, since we assume a homogeneous diffusion of the drug. Free phages are randomly re-assorted to new positions if there are fewer phages than the total number of locations. Otherwise, a uniform re-distribution, or as uniform as possible (i.e., if after a uniform distribution of the majority of the phages, the remaining ones, if any, are allocated randomly to a location), of the phage for all the locations is performed. This allows a homogeneous distribution of the phage throughout the environment.

For semi-solid environments, both antibiotics and phage are locally, but randomly, switched amongst locations within Moore distance 3.

In environments that are spatially structured, phages can only diffuse between cells upon infecting a bacterial cell, whilst antibiotics do not diffuse.

#### Antibiotic placement in spatially structured environments

The arrangement of antibiotics in spatially structured environments is defined by the parameter **Antibiotic Arrangement in Structured Environments**. If this is set as “Homogeneous”, antibiotics are deployed as in well-mixed environments (each location harbours the same antibiotic concentration). On the other hand, “Heterogeneous” antibiotic arrangements create stochastic landscapes of antibiotic concentrations across the environment in the following way:

- First, a random sample (with replacement) of half of all locations is performed.

- Second, an antibiotic concentration (with the value randomly chosen between the maximum antibiotic concentration defined and half of this value) is deposited in each chosen location.

- Third, diffusion occurs recursively for each location with antibiotics, with the diffused antibiotic concentration that reaches a new location being randomly chosen between X and X/2 (with X=exponential decayed (with lambda 0.17) antibiotic concentration from the location where the diffusion originates).

- Finally, the recursive diffusion occurs while the antibiotic concentration in the new location is above 1.

## REFERENCES

1. Werisch, M., Berger, U. & Berendonk, T. U. Conjugative plasmids enable the maintenance of low cost non-transmissible plasmids. *Plasmid* **91,** 96–104 (2017).

2. Haerter, J. O., Trusina, A. & Sneppen, K. Targeted bacterial immunity buffers phage diversity. *J. Virol.* **85,** 10554–10560 (2011).

3. Zeng, L. *et al.* Decision making at a subcellular level determines the outcome of bacteriophage infection. *Cell* **141,** 682–691 (2010).

4. Weitz, J. S., Hartman, H. & Levin, S. A. Coevolutionary arms races between bacteria and bacteriophage. *Proc. Natl. Acad. Sci. U.S.A.* **102,** 9535–9540 (2005).

5. Lamont, I., Brumby, A. M. & Egan, J. B. UV induction of coliphage 186: prophage induction as an SOS function. *Proc. Natl. Acad. Sci. U.S.A.* **86,** 5492–5496 (1989).

6. Kourilsky, P. Lysogenization by bacteriophage lambda. I. Multiple infection and the lysogenic response. *Mol. Gen. Genet.* **122,** 183–195 (1973).

7. St-Pierre, F. & Endy, D. Determination of cell fate selection during phage lambda infection. *Proc. Natl. Acad. Sci. U.S.A.* **105,** 20705–20710 (2008).

8. Erez, Z. *et al.* Communication between viruses guides lysis-lysogeny decisions. *Nature* **541,** 488–493 (2017).

9. Susskind, M. M., Botstein, D. & Wright, A. Superinfection exclusion by P22 prophage in lysogens of Salmonella typhimurium. III. Failure of superinfecting phage DNA to enter sieA+ lysogens. *Virology* **62,** 350–366 (1974).

10. Bondy-Denomy, J. *et al.* Prophages mediate defense against phage infection through diverse mechanisms. *ISME J* **10,** 2854–2866 (2016).

11. Ackermann, M. A functional perspective on phenotypic heterogeneity in microorganisms. *Nat Rev Micro* **13,** 497–508 (2015).

12. Golding, I. Infection by bacteriophage lambda: an evolving paradigm for cellular individuality. *Curr Opin Microbiol* **43,** 9–13 (2018).

13. Kohanski, M. A., Dwyer, D. J. & Collins, J. J. How antibiotics kill bacteria: from targets to networks. *Nat Rev Micro* **8,** 423–435 (2010).

14. Davies, J., Spiegelman, G. B. & Yim, G. The world of subinhibitory antibiotic concentrations. *Curr Opin Microbiol* **9,** 445–453 (2006).

15. Ackermann, M., Stearns, S. C. & Jenal, U. Senescence in a bacterium with asymmetric division. *Science* **300,** 1920–1920 (2003).

16. Demidenko, E., Glaholt, S. P., Kyker-Snowman, E., Shaw, J. R. & Chen, C. Y. Single toxin dose-response models revisited. *Toxicol. Appl. Pharmacol.* **314,** 12–23 (2017).

17. Servais, P., Billen, G. & Rego, J. V. Rate of bacterial mortality in aquatic environments. *Appl. Environ. Microbiol.* **49,** 1448–1454 (1985).

18. Liu, Y. Q., Zhang, Y. Z. & Gao, P. J. Novel concentration-killing curve method for estimation of bactericidal potency of antibiotics in an in vitro dynamic model. *Antimicrob Agents Chemother* **48,** 3884–3891 (2004).

19. Levin, B. R. & Udekwu, K. I. Population dynamics of antibiotic treatment: a mathematical model and hypotheses for time-kill and continuous-culture experiments. *Antimicrob Agents Chemother* **54,** 3414–3426 (2010).

20. Nanda, A. M., Thormann, K. & Frunzke, J. Impact of spontaneous prophage induction on the fitness of bacterial populations and host-microbe interactions. *J Bacteriol* **197,** 410–419 (2015).

21. Oppenheim, A. B., Kobiler, O., Stavans, J., Court, D. L. & Adhya, S. Switches in bacteriophage lambda development. *Annu. Rev. Genet.* **39,** 409–429 (2005).

22. Castellazzi, M., George, J. & Buttin, G. Prophage induction and cell division in E. coli. II. Linked (recA, zab) and unlinked (lex) suppressors of tif-1-mediated induction and filamentation. *Mol. Gen. Genet.* **119,** 153–174 (1972).

23. Touchon, M., Moura de Sousa, J. A. & Rocha, E. P. Embracing the enemy: the diversification of microbial gene repertoires by phage-mediated horizontal gene transfer. *Curr Opin Microbiol* **38,** 66–73 (2017).

24. Ikeda, H. & Tomizawa, J. I. Transducing fragments in generalized transduction by phage P1. 3. Studies with small phage particles. *J. Mol. Biol.* **14,** 120–129 (1965).

25. Drake, J. W. Comparative rates of spontaneous mutation. *Nature* **221,** 1132 (1969).

26. Meyer, J. R. *et al.* Repeatability and contingency in the evolution of a key innovation in phage lambda. *Science* **335,** 428–432 (2012).

27. Semenova, E. *et al.* Interference by clustered regularly interspaced short palindromic repeat (CRISPR) RNA is governed by a seed sequence. *Proc. Natl. Acad. Sci. U.S.A.* **108,** 10098–10103 (2011).

28. Simmons, M., Drescher, K., Nadell, C. D. & Bucci, V. Phage mobility is a core determinant of phage-bacteria coexistence in biofilms. *ISME J* **89,** 135 (2017).

29. Labrie, S. J., Samson, J. E. & Moineau, S. Bacteriophage resistance mechanisms. *Nat Rev Micro* **8,** 317–327 (2010).

30. Pan, M. & Chu, L. M. Adsorption and degradation of five selected antibiotics in agricultural soil. *Sci. Total Environ.* **545-546,** 48–56 (2016).

31. De Paepe, M. & Taddei, F. Viruses' life history: towards a mechanistic basis of a trade-off between survival and reproduction among phages. *PLoS Biol.* **4,** e193 (2006).

# Parameters used in the main analyses

For information on the effects of parameters, see the ODD.

Table 2.1. Description of the populations used in the analyses in the main text.

| Population | Experiments concerned | #initial cells |
| --- | --- | --- |
| B1 (Phase 1) | All | 5000 |
| B2 (Phase 2) | All | 5000 |
| Phage A (Phase 1) | 1,4 and 5 | 100 |
| Phage B (Phase 1) | 2 and 4 | 100 |
| Phage C (Phase 1) | 3 and 5 | 100 |

Table 2.2. General parameters used in the simulations.

| Variable | Value |
| --- | --- |
| Maximum bacteria | 10000 |
| Phage degradation lambda | 0.015 |
| Antibiotic degradation lambda | 0.0001 |
| Maximum antibioic concentration | 30 |
| Phage particles sampled (phase 1 ---> phase 2) | 10000 |
| Number of bacterial genes | 30 |
| Death curve (A, B, M) | 0, 0.08, 27 |
| Antibioic resistance mutation rate | 0 |
| Antibiotic resistance cost | 0.1 |
| Phage resistance mutation rate | 0 |
| Phage resistance cost | 0.1 |
| Phage burst probability | 0.4 |
| Infection distance | 2 |

Table 2.3. Basal growth rates of populations (can be modulated by costs of resistance).

| Bacteria Population | Growth Rate |
| --- | --- |
| B1 | 0.2 |
| B2 | 0.2 |

Table 2.4. Variables of phage populations.

| Phage Population | Burst Size | Generalized Transduction Probability | Maximum Genes In Cargo | Lysogeny (A, K) | Induction (A, K) | Specialized Transduction Probability |
| --- | --- | --- | --- | --- | --- | --- |
| Phage A | 50 | 0.01 | 10 | 100,0.05 | 100000/1000*, 0.4 | 0 |
| Phage B | 50 | 0.01 | 10 | 10E200, 0.05** | 100000, 0.4** | 0** |
| Phage C | 50 | 0 | 10 | 100,0.05 | 100000, 0.4 | 0 |

| * Changed for high induction variant of Figure S3C |
| --- |
| ** Not applicable to virulent phage |

Table 2.5. Phage-host attachment probability matrix. All phages have the same attachment probability to bacteria in all simulations.

| Bacteria | B1 | B2 |
| --- | --- | --- |
| Phage A | 0.01 | 0.01 |
| Phage B | 0.01 | 0.01 |
| Phage C | 0.01 | 0.01 |

# Parameters used in the Random forest analysis

The random forest analysis involves the simulation of the model using random combinations of parameters. Some parameters are fixed (Table 3.1), whereas the others are varied (Table 3.2). For the latter, we tested a varied range of parameters between low and high values for the variable. Their effects are represented in the figures at the end of the section.

Table 3.1 – Parameters that were fixed in the Random Forest Analysis.

| Fixed Parameters | Value |
| --- | --- |
| Iterations infection period | 10 |
| Iterations transduction period | 100 |
| Antibiotic iteration (transduction period) | 7 |
| World Size | 100x100 (10^4^ bacteria) |
| World Type | Toroidal |
| Maximum Antibiotic Concentration | 30 |
| Antibiotic Degradation Lambda | 0.0001 |
| Number of genes | 30 |
| Phage Carrying Capacity (per grid location) | 500 |
| Phage Resistant Receptor Cost | 0.1 |
| ARG Cost | 0.1 |
| Environmental diffusion | Well Mixed |
| Phage maximum capacity (in bacterial genes) | 10 |
| Resistance Phage CRISPR Mutation Probability | 0 |
| Abortive Infection Probability | 0 |
| Curing Probability | 0 |
| Specialized transduction probability | 0 |
| Phage Receptor Mutation probability | 0 |

Table 3.2. Parameters that were varied with the Random Forest Analysis.

| **Variable Parameters** | **Low** | **Middle** | **High** | **Very High** | **Comments** |
| --- | --- | --- | --- | --- | --- |
| Bacterial Growth Rate | 0.1 | 0.2 | 0.5 | NA | Probability of division (per iteration) of both antibiotic resistant and antibiotic sensitive bacteria. See Figure 3.1 below, showing bacteria growing in the absence of antibiotics or phage. The range of this variable leads to filling the lattice in a broad range of times. |
| Phage Degradation Lambda | 0.01 | 0.015 | 0.03 | NA | Parameter used for calculation of probability of a single phage particle to disappear from the environment (decay, see ODD for mathematical equation). See Figure 3.2 below, showing phage (population) decay in the absence of hosts to infect (elimination of most phages varies in time by a factor of 2). |
| Phage Burst Size | 20 | 50 | 80 | NA | Typical values for dsDNA temperate phages, Calendar's book indicates: 50-200. Cyanophages: 40-80. Lambda = 150, P1 = 100-200. |
| Burst Probability (after induction/infection) | 0.2 | 0.4 | 0.7 | NA | This produces a delay in burst after induction. In the lowest case it’s similar to doubling time (Middle value) and in the highest case it’s much lower than a bacterial doubling time. |
| Bacterial Death Curve (A, B, M) | NA | (0, 0.08, 27) | (0.1, 0.2, 25) | NA | See Figure 3.3 below, showing the probability of death relative to antibiotic concentration (see ODD) for the dynamics of death given by each parameter combination |
| Resistance Phage Receptor Mutation Probability | 0 | 10^-7^ | 10^-5^ | NA | Thirion (Genetics, 72) describes frequency of E. coli mutants resistant to lambda at frequencies between 10^-6 and 10^-8, scaled at 0.2 generation rate, this gives values close to the medium and max |
| ARG Mutation Probability | 0 | 10^-7^ | 10^-5^ | NA | Probability of acquiring a mutation allowing to resist to antibiotics is between zero and a value that is much higher than expected given bacterial mutation rates (often ~10^-10), which means that there would have to be 10^5 sites that by mutation would lead to resistance. |
| Generalized transduction probability | 0 | 10^-3^ | 10^-2^ | 10^-1^ | Probability was set high to accommodate the small population size. The highest value corresponds to values obtained by Kenzaka, ISMEj, 2010. |
| Phage Inoculum (infection period) | 50 | 100 | 500 | NA | Number of inoculated phage that will infect antibiotic resistant bacteria at the initial stage |
| **Host infection (adsorption probability)** | 0.01 | 0.1 | 0.8 | NA | Probability of a single viral particle to infect a host bacteria upon their encounter in the environment. See Figure 3.4 below, showing the adsorption curves (population) relative to each individual adsorption probability value. Phages were introduced at iteration 7, when bacteria reach maximum population size, and the number of free phage particles was assayed in the following iteration. This range of parameters allows to obtain a wide range of effects after one iteration, from a small frequency of adsorption to almost complete adsorption. |
| **Infection Distance** | 0 | 2 | 4 | NA | Phages must be in the cell of the lattice for infection, or can be quite further away. |
| **Lysogeny Probability Alpha/Kappa** | (10^3^, 0.1) | (10^2^, 0.1) | (10^5^, 0.1) | NA | Parameters used for the calculation of the probability of lysogenization upon infection, relative to the concentration of nearby phage particles (see ODD). Figure 3.5 below shows the probability of lysogenization relative to each parameter combination |
| **Induction Probability Alpha/Kappa** | (10^3^, 0.1) | (10^3^, 0.25) | (10^3^, 0.6) | NA | Parameters used for the calculation of the probability of prophage induction, relative to the concentration of antibiotics. Parameters define also the spontaneous induction in the absence of antibiotics (see ODD). For these parameters, spontaneous induction is the same across the 3 conditions. See Figure 3.6 below for the probability of induction for each parameter combination. This range of parameters provides a range of outcomes where small concentration induces most cells in one iteration to a range where only near maximal conditions does so. |
| **Sampled Phage (infection period -> transduction period)** | 5000 | 10000 | 15000 | NA | Number of phage particles that are sampled after infection of antibiotic resistant bacteria, and before the infection of antibiotic sensitive bacteria. Includes both active phage particles and transducing particles |


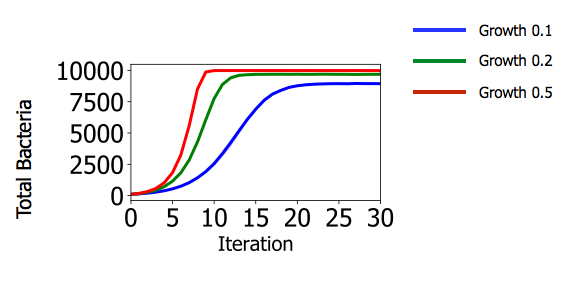


Figure 3.1. Time required to fill the lattice in function of the different bacterial growth rates.


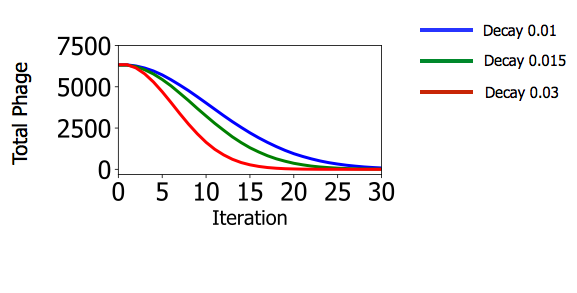


Figure 3.2. Number of phages in the population in function of the phage decay function.

Figure 3.3. Change in the probability of death per iteration in function of antibiotic concentration.


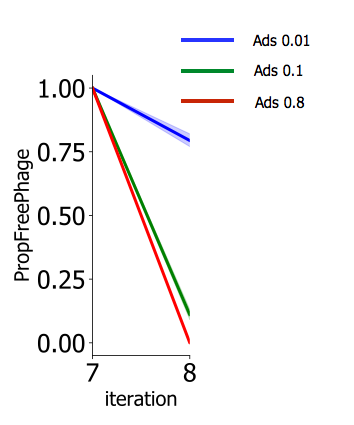


Figure 3.4. Proportion of free phage between two iterations for different adsorption rates.

Figure 3.5. Probability of entering the lysogen state upon infection in relation the number of phage for the three different lysogeny functions.

Figure 3.6. Probability of death by phage induction per iteration in function of antibiotic concentration.
