# Supplementary figures and images for: Bacteriophages benefit from generalized transduction
Source: PLoS Pathog. 2019 Jul 5;15(7):e1007888. doi: 10.1371/journal.ppat.1007888 (PMC6636781; doi:10.1371/journal.ppat.1007888)

**Supplementary Figure S1**


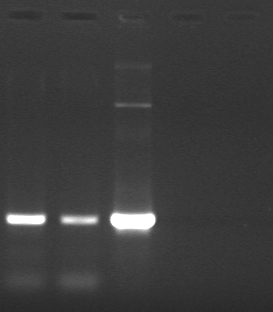

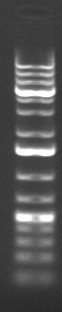


1 2 3 4 5

Supplement: S1 Fig — Product of PCR reactions performed with pRMC2 specific primers and DNA isolated from 80α-vir phage lysate (lane1), from Φsa012 phage lysate (lane 2), pRMC2 plasmid preparation (lane 3), 80α-vir phage lysate prior to DNA purification (lane 4) and from Φsa012 phage lysate prior to DNA purification (lane 5). (DOCX) [file ppat.1007888.s005.docx]

**Supplementary figure S4**

**
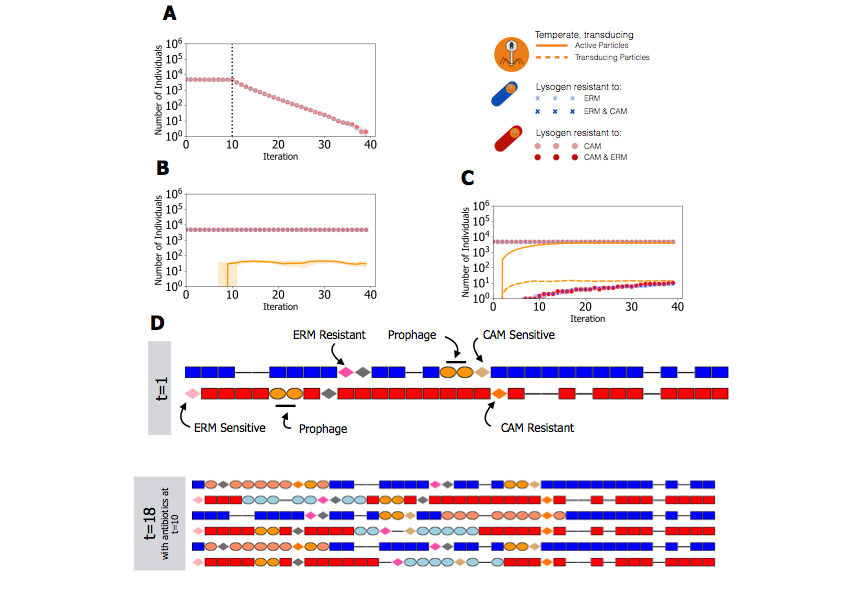
**

Supplement: S4 Fig — Setup of the simulations is similar to the one in Fig 6A. A) Population dynamics with non-lysogenic bacteria subjected to antibiotic exposure. B and C) Population dynamics with lysogenic bacteria in the absence of antibiotics, with different parameterizations of the model’s induction function (alpha = 100000, kappa = 0.4, equivalent to spontaneous induction rate of 10−5, for B; alpha = 1000, kappa = 0.4, equivalent to spontaneous induction rate of 10−3, for C). Lines correspond to the median of 100 different simulations with similar parameters, and the shaded areas correspond to a confidence interval of 95%. E) sample of the genomic composition of populations in Fig 6B at the beginning (t = 1) and sometime after antibiotic exposure (t = 18) of a typical simulation. Symbols are as explained in S2 Fig. CAM resistance genes are shown in orange (bright orange for resistant phenotype, dimmed orange for sensitive phenotype), whilst ERM resistance genes are shown in pink (bright pink for resistant phenotype, dimmed pink for sensitive phenotype). (DOCX) [file ppat.1007888.s008.docx]

**Supplementary figure S5**


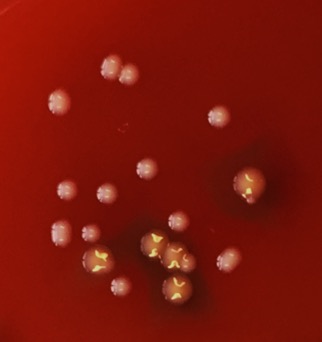
**
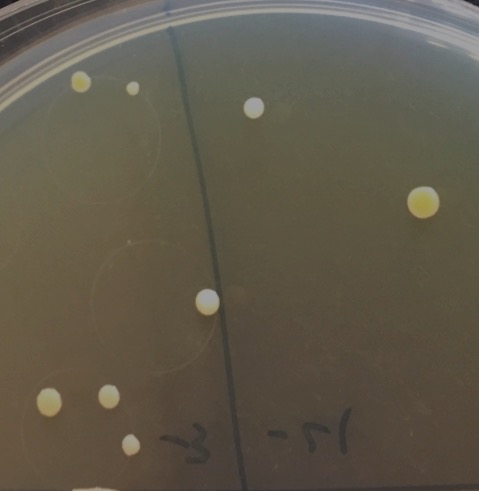
**

Supplement: S5 Fig — Mixed culture of AA001 and AA002 grown on TSA (A) or TSA supplemented with 5% blood (B). AA001 (8325–4 background) appears white and non-hemolytic whereas AA002 (LAC background) appears yellow and hemolysis-positive. (DOCX) [file ppat.1007888.s009.docx]
