# Supplementary material for: Bacteriophages benefit from generalized transduction
Source: PLoS Pathog. 2019 Jul 5;15(7):e1007888. doi: 10.1371/journal.ppat.1007888 (PMC6636781; doi:10.1371/journal.ppat.1007888)
Supplement: S3 Fig — A-B) Each dot corresponds to a single parameter combination (5000 in total). Each simulation was repetead 20 times, and the fraction of simulations that show phage survival (number of phage particles in the environment or prophages within bacterial genomes) are shown in the y-axis. A) Outcome relative to different values of probability of generalized transduction explored (x-axis) when the probability of lysogeny was “high” (see parameters in section 3 of Supplementary text file S1). B) Outcome relative to different values of probability of generalized transduction explored (x-axis) when the probability of lysogeny was “Middle” (see parameters in section 3 of Supplementary text file S1). This shows the impact of the frequency of generalized of transduction as long as there is some level of lysogenization, in spite of the extensive variation of the remaining parameters in these simulations. (DOCX) [file ppat.1007888.s007.docx]

**Supplementary figure S3**
