# Supplementary material for: Bacteriophages benefit from generalized transduction
Source: PLoS Pathog. 2019 Jul 5;15(7):e1007888. doi: 10.1371/journal.ppat.1007888 (PMC6636781; doi:10.1371/journal.ppat.1007888)
Supplement: S6 Fig — Spontaneous and mitomycin C induced phage release from 8325-4-ϕ11 (AA001; p<0.05, Student t-test) and LAC- ϕ11 (AA002) (p<0.01, Student t-test) monocultures as well as mixed cultures of the two strains. (DOCX) [file ppat.1007888.s010.docx]

**Supplementary figure S6**
